# Discovery of Tumor-Targeted 6-Methyl Substituted Pemetrexed and Related Antifolates with Selective Loss of RFC Transport

**DOI:** 10.1021/acsmedchemlett.3c00326

**Published:** 2023-11-15

**Authors:** Krishna Kaku, Manasa P. Ravindra, Nian Tong, Shruti Choudhary, Xinxin Li, Jianming Yu, Mohammad Karim, Madelyn Brzezinski, Carrie O’Connor, Zhanjun Hou, Larry H. Matherly, Aleem Gangjee

**Affiliations:** † Division of Medicinal Chemistry, Graduate School of Pharmaceutical Sciences, Duquesne University, 600 Forbes Avenue, Pittsburgh, Pennsylvania 15282, United States; ‡ Molecular Therapeutics Program, Barbara Ann Karmanos Cancer Institute, 4100 John R, Detroit, Michigan 48201, United States; § Department of Oncology, Wayne State University School of Medicine, Detroit, Michigan 48201, United States; ∥ Department of Pharmacology, Wayne State University School of Medicine, Detroit, Michigan 48201, United States

**Keywords:** Antifolate, Folate receptor, Pemetrexed, Proton-coupled folate transporter, Reduced folate carrier

## Abstract

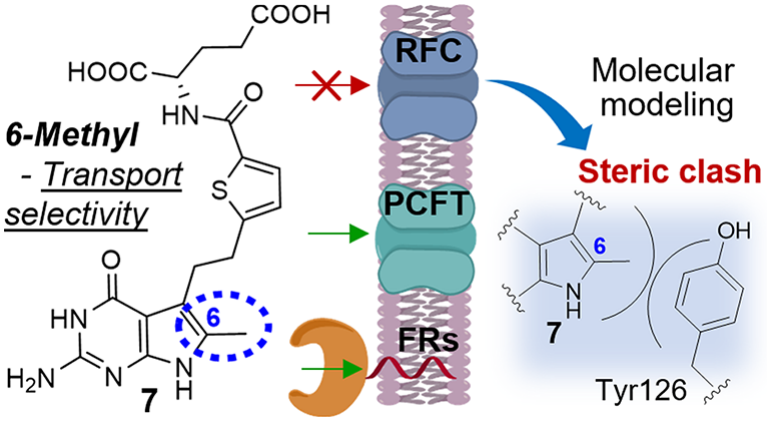

Pemetrexed and related 5-substituted pyrrolo[2,3-*d*]pyrimidine antifolates are substrates for the ubiquitously expressed reduced folate carrier (RFC), and the proton-coupled folate transporter (PCFT) and folate receptors (FRs) which are more tumor-selective. A long-standing goal has been to discover tumor-targeted therapeutics that draw from one-carbon metabolic vulnerabilities of cancer cells and are selective for transport by FRs and PCFT over RFC. We discovered that a methyl group at the 6-position of the pyrrole ring in the bicyclic scaffold of 5-substituted 2-amino-4-oxo-pyrrolo[2,3-*d*]pyrimidine antifolates **1**–**4** (including pemetrexed) abolished transport by RFC with modest impacts on FRs or PCFT. From molecular modeling, loss of RFC transport involves steric repulsion in the scaffold binding site due to the 6-methyl moiety. 6-Methyl substitution preserved antiproliferative activities toward human tumor cells (KB, IGROV3) with selectivity over IOSE 7576 normal ovary cells and inhibition of *de novo* purine biosynthesis. Thus, adding a 6-methyl moiety to 5-substituted pyrrolo[2,3-*d*]pyrimidine antifolates affords tumor transport selectivity while preserving antitumor efficacy.

One-carbon (C1) metabolism offers an important therapeutic target for many cancers, reflecting its role in the biosynthesis of purines, thymidylate, serine, and methionine, and in supporting biological methylation reactions from S-adenosylmethione.^1^ Chemotherapy with antifolates has been a cornerstone of cancer therapy for over 60 years.^2^ Clinically used antifolates include methotrexate (MTX), pemetrexed (PMX), pralatrexate (PTX), and raltitrexed (RTX) (Figure 1). While antifolates serve important roles in the therapeutic armamentarium for cancer, they manifest clinical challenges, most notably dose-limiting toxicities and emergence of drug resistance.^2^

**Figure 1:**
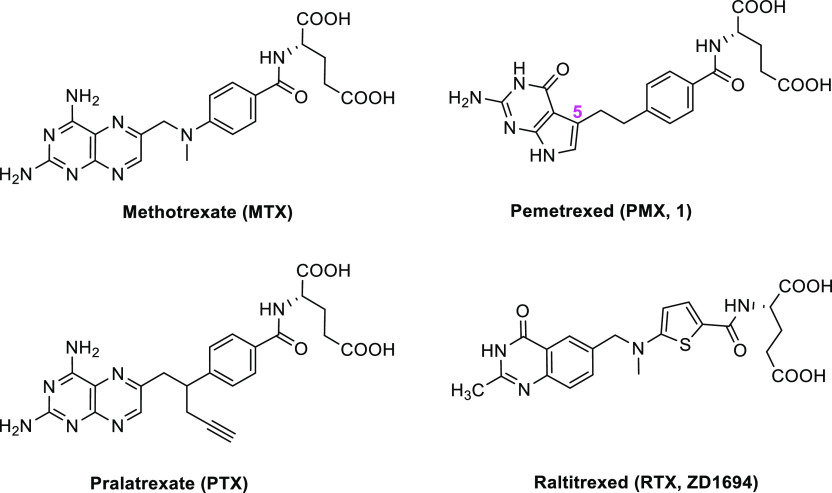
Clinically used antifolates.

Membrane transport of antifolates is integral to their therapeutic efficacy in treating a variety of malignancies and nonmalignant conditions.^2,3^ Pharmacologically important folate transporters include the reduced folate carrier (RFC) (SLC19A1), the proton-coupled folate transporter (PCFT) (SLC46A1), and the high-affinity folate receptor (FR) α and FRβ.^4−8^ These systems mediate cellular accumulations of folate cofactors and classic antifolates; however, they have different substrate specificities and mechanisms.^3,5,7−9^ Both RFC and PCFT are facilitative transporters; RFC is a folate-organic anion antiporter,^5^ and PCFT is a folate-proton symporter.^7^ FRs are glycosyl phosphatidylinositol-modified proteins that mediate cellular uptake of (anti)folates by receptor-mediated endocytosis.^4,10^

RFC is ubiquitously expressed and is the major tissue transporter of folates.^5^ Dose-limiting toxicities of classic antifolates are partially due to their membrane transport by RFC in both normal tissues and tumors.^5^ In addition, loss of transport in tumors due to decreased expression or mutation of RFC results in insufficient levels of intracellular antifolates to inhibit metabolic targets and sustain polyglutamate synthesis.^5,11^

PCFT is the major mechanism for intestinal folate uptake.^12^ Although PCFT is expressed in a select number of other tissues including the choroid plexus, kidney, liver, and spleen,^13,14^ PCFT is not a major tissue transporter of folates. Further, transport by PCFT is optimal at acidic pHs not commonly associated with most tissues,^13,15^ and the modest level of PCFT transport that occurs at neutral pH is suppressed by bicarbonate.^16^ Notably, PCFT is expressed in solid tumors including malignant pleural mesothelioma,^17^ pancreatic adenocarcinoma,^18^ nonsmall cell lung cancer,^19^ and epithelial ovarian cancer^20^ and is highly active in the acidic tumor microenvironment.^6,13^

FRα is expressed in normal epithelial tissues such as kidney, lung, choroid plexus, and placenta and in several tumors including ovarian cancer, nonsmall cell lung cancer, triple negative breast cancer, and kidney, endometrial, and colorectal cancers.^4,9,21^ However, only FRα in tumors exhibits the basolateral exposure required for access to the circulation.^4^ FRβ is expressed in placenta, mature neutrophils, and activated monocytes and macrophages, along with acute myeloid leukemia (AML) blasts.^4,9^

PMX (**1**) is a 5-substituted pyrrolo[2,3-*d*]pyrimidine benzoyl antifolate with a 2-carbon bridge (Figure 1). PMX primarily inhibits thymidylate synthase (TS) with secondary inhibition at glycinamide ribonucleotide formyltransferase (GARFTase) and 5-aminoimidazole-4-carboxamide (AICA) ribonucleotide formyl transferase (AICARFTase) in *de novo* purine biosynthesis.^22^ The 5-substituted pyrrolo[2,3-*d*]pyrimidine benozyl compound **2** (3-carbon bridge) and the analogous pyrrolo[2,3-*d*]pyrimidine thienoyl compound **3** (2-carbon bridge) (Figure 2) were reported to inhibit GARFTase and AICARFTase.^23,24^ While these compounds would presumably circumvent resistance due to alterations in TS, the 5-substituted pyrrolo[2,3-*d*]pyrimidine compounds including PMX all exhibit promiscuous transport in that they are substrates for RFC as well as for PCFT and FRs.^23,24^

**Figure 2:**
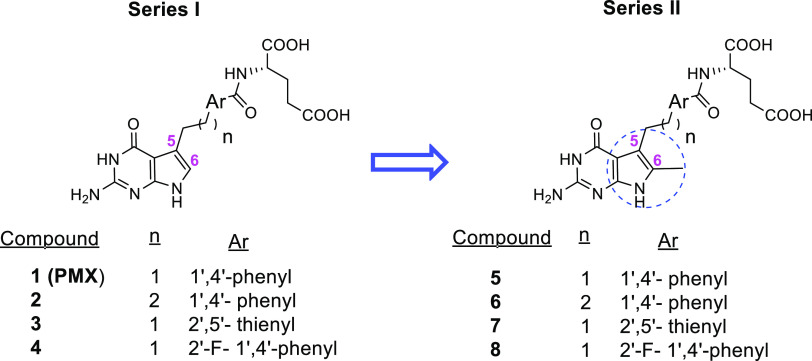
6-Unsubstituted lead compounds (Series I) and the corresponding 6-methyl analogues (Series II).

Our long-standing goal has been to discover potent folate-based, tumor-targeted agents that draw from unique C1 metabolic vulnerabilities of cancer cells and are selective for membrane transport by FRs and PCFT over RFC.^13,19,25−31^ We envisage that cytotoxic C1 inhibitors that are selectively transported by PCFT or FRs with limited transport by RFC would be far less toxic to normal tissues and would also circumvent resistance due to loss of RFC.

We reasoned that if a simple structural modification could be introduced into targeted antifolates to abolish RFC transport, while preserving uptake by FRs and/or PCFT along with intracellular target engagement, a more effective agent would be realized. One such structural modification involves adding a 6-methyl group to the 5-subsituted pyrrolo[2,3-*d*]pyrimidine scaffold. We hypothesized that a strategically placed methyl moiety could sterically hinder drug attachment and net transport by RFC. As long as the net impact was greater toward RFC than on either PCFT or FRs, this would result in a more tumor-selective analogue than the corresponding nonmethylated compound. In addition to a potential impact on substrate binding to RFC, 6-methyl substitution also effects conformational restriction on the flexibility of the 5-substituted pyrrolo[2,3-*d*]pyrimidine compounds; this could further enhance selectivity for transport by PCFT and FRs over RFC as well as target engagement.

In this report, we extend our systematic structure–activity relationship (SAR) studies of pyrrolo[2,3-*d*]pyrimidine antifolates related to PMX (**1**) by introducing a methyl group at the unsubstituted 6-position of the pyrrole ring in the bicyclic scaffolds of 5-substituted pyrrolo[2,3-*d*]pyrimidine antifolates **1**–**4**, generating **5**–**8**, respectively (Figure 2). Compound **4**, the fluorinated analogue of PMX (**1**) and compound **5**, the 6-methyl analogue of PMX (**1**), were published previously;^32,33^ however, there is no report in the literature regarding their transport properties.

Given our goal to test the impact of 6-methyl substitutions on 5-substituted pyrrolo[2,3-*d*]pyrimidine antifolates **1**–**4** on cellular uptake by the pharmacologically important folate transporters RFC, PCFT, and FRs, it was important to model the proposed 6-methyl-substituted analogues compared to the corresponding des-methyl compounds in the known structures for PCFT (PDB: 7BC7),^21,34^ FRα (PDB: 5IZQ),^35^ FRβ (PDB: 4KN2),^36^ and RFC (PDB: 8GOF).^37^ PMX (**1**) and its 6-methyl analogue (**5**) were selected as representative of the four pairs of des-methyl (**1**–**4**) and 6-methyl compounds (**5**–**8**) (**Series I and II**, respectively; Figure 2) for the computational study.

In Figure 3, compounds **1** (green) and **5** (purple) and the residues within 5 to 6 Å of the C6 position are shown as space-filled models. The folate binding sites of PCFT (Figure 3a,b), FRα (Figure 3c,d), and FRβ (Figure 3e,f) can all accommodate a 6-methyl group, without any steric hindrance. However, in the binding site for RFC (Figure 3g,h), the 6-methyl group of **5** (**panel h**) causes a serious steric clash with Tyr126, while breaking the edge-to-face π interaction of the pyrrole ring with Tyr126. Thus, the 6-methyl moiety of compound **5** seems likely to interfere with cellular uptake by RFC, although the 6-methyl group does not appear to compromise the binding and transport of **5** by PCFT or FRs α or β. Similar simulations with compounds **6**–**8** provided identical conclusions (see Figure S1 in the Supporting Information).

**Figure 3:**
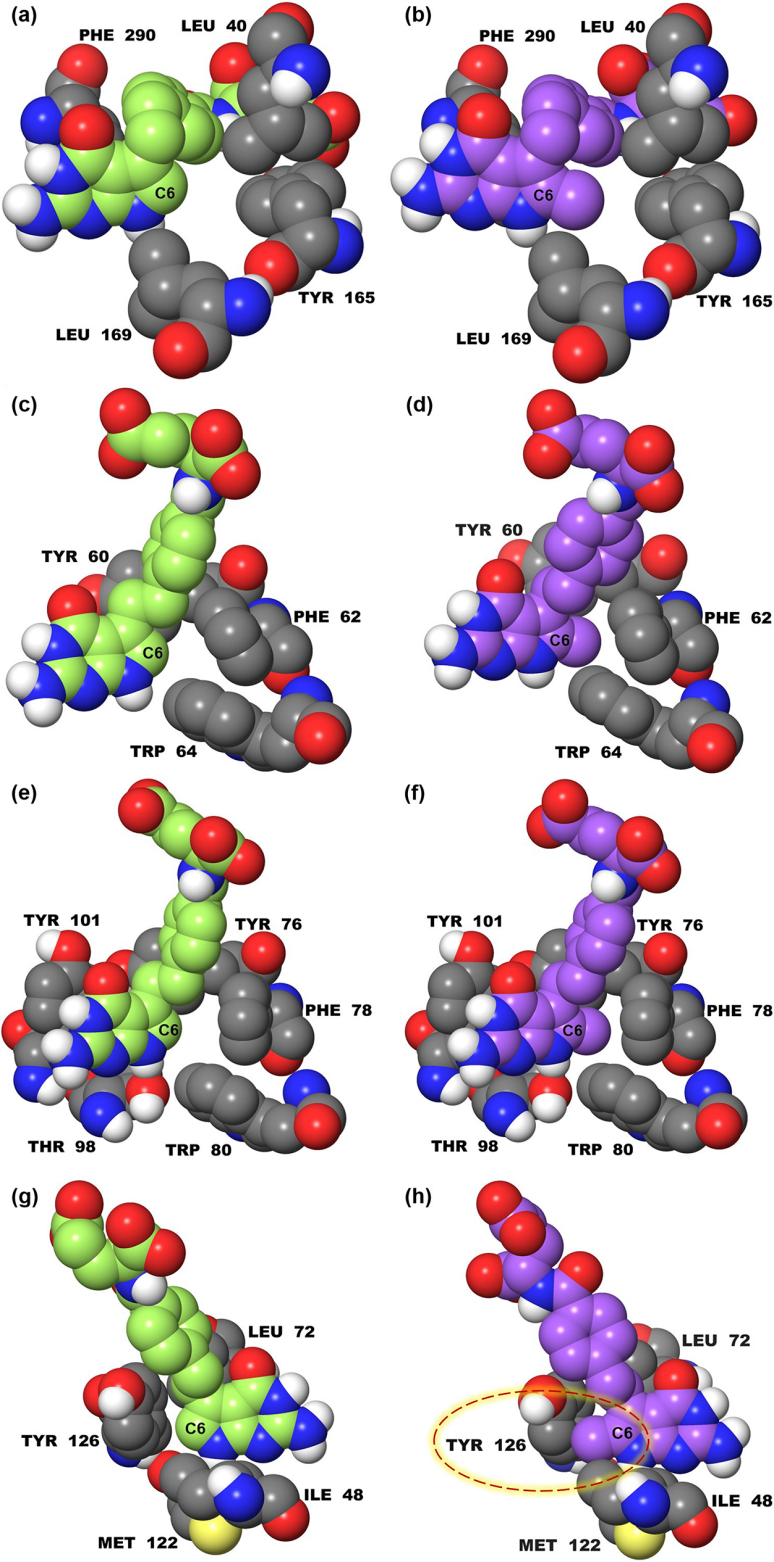
**Docked poses of 1 (PMX, green) and 5 (6-methyl PMX analogue, purple) in binding pockets of (a, b) PCFT, (c, d) FRα, (e, f) FRβ, and (g, h) RFC.** The ligands in (g, h) are shown 180° horizontally flipped compared to (a–f) for a better view in the RFC binding pocket.

To assess the 6-methyl moiety’s ability to restrict movement of the flexible 5-substituent compared to the 6-unsubstituted analogues, we performed a low-mode conformational search (LMCS) using MacroModel.^38^ Conformations within 5 kcal/mol of the lowest-energy conformer were generated. The conformation numbers obtained for each of the energy-minimized compounds (in parentheses) are as follows: **1** (268) vs **5** (243); **2** (605) vs **6** (535); **3** (359) vs **7** (281); and **4** (339) vs **8** (299). Thus, compared to the parent 6-des-methyl pyrrolo[2,3-*d*]pyrimidine compounds **1**–**4**, the corresponding 6-methyl compounds **5**–**8** exhibit substantially fewer conformations.

For the synthesis, nucleophilic aromatic substitution of the commercially available 2-amino-6-chloropyrimidin-4(3*H*)-one **9** (Scheme 1) with hydrazine hydrate afforded the intermediate hydrazine **10**.^32^

**Scheme 1:**
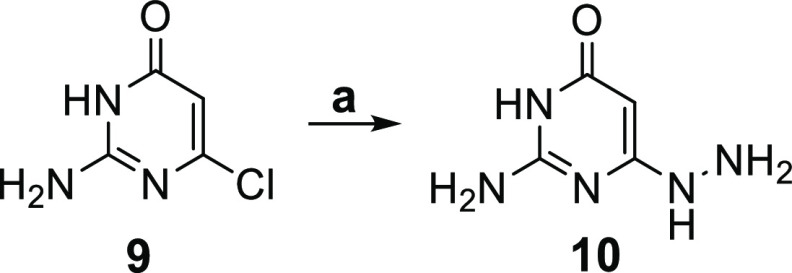
Reagents and conditions: (a) Hydrazine hydrate, H_2_O, reflux, 3 h, 72%.

Palladium-catalyzed Heck coupling of commercially available starting materials **11a** and **11c** (Scheme 2) and alkenol **12** gave the coupled, unsaturated secondary alcohols that rearranged to the vinyl alcohols and tautomerized to afford the ketones **17a** and **17c**. A similar Heck coupling of **11a** with alkenone **13** provided an unsaturated ketone **14** which underwent catalytic hydrogenation to give **17b**. The ketone intermediate **17d** was synthesized using a modified Heck coupling reaction between **11d** and alkenol **12**, employing an electron-rich phosphine ligand.

**Scheme 2:**
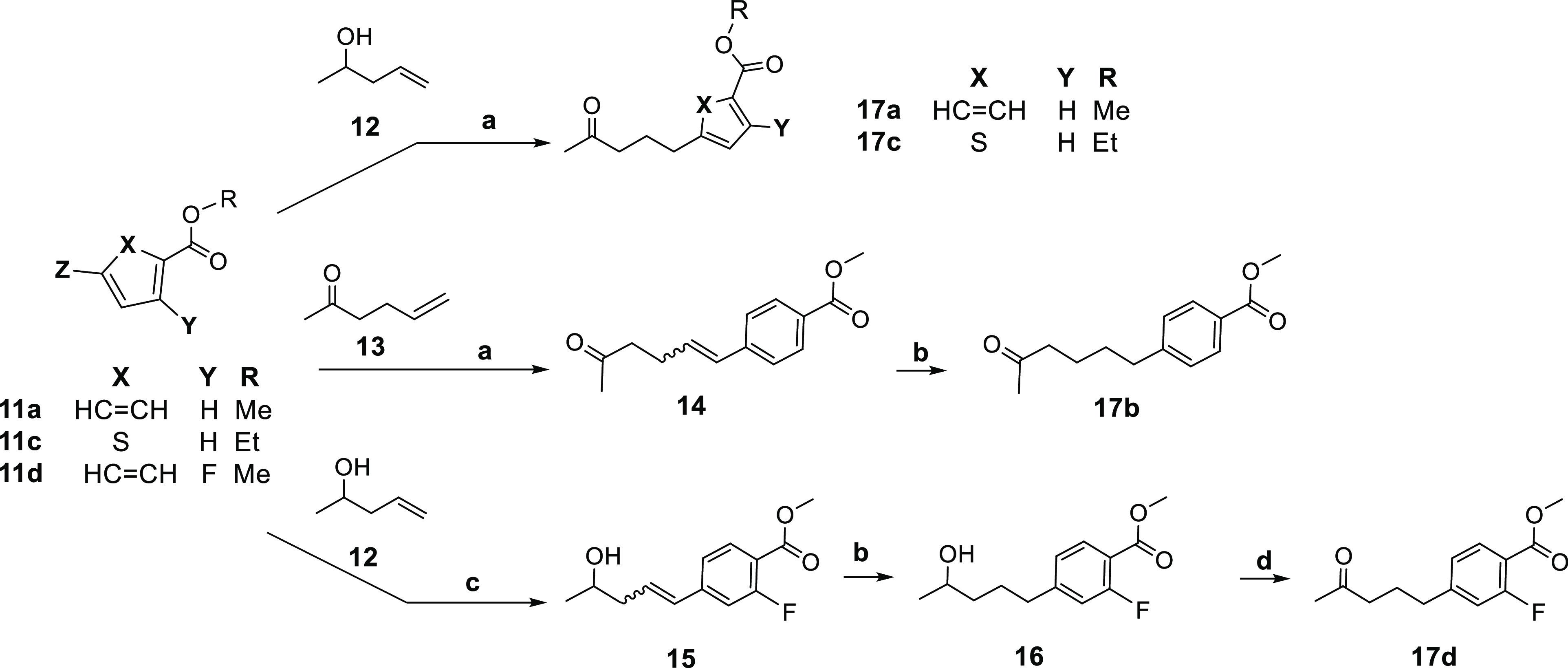
Reagents and conditions: (a) Bu_4_NCl, Pd(OAc)_2_, LiCl, LiOAc, or KOAc, DMF, 80 °C, 3–16 h, 64–97% (Z = I or Br); (b) 5% Pd/C, H_2_, 35 psi, rt, 24 h, 90–95%; (c) 2-(di-*tert*-butylphosphino)-1-phenylindole, Pd(dba)_2_, TEA, 80 °C, 2 h, 52%; (d) DMP, CHCl_3_, 3 h, rt, 67%.

A key step in the synthesis of the 5-substituted 2-amino-4-oxo-6-methyl pyrrolo[2,3-*d*]pyrimidine classical antifolates involved thermal indolization of the hydrazones **18a**–**d**^32^ (Scheme 3). Condensation of the ketones **17a**–**d** with the hydrazine **10** under reflux in 2-methoxyethanol afforded the key pyrimidine hydrazone intermediates **18a**–**d**.^32^ Fischer indole cyclization of hydrazones **18a**–**d** was accomplished regioselectively to the 5-substituted, 2-amino-4-oxo-6-methyl pyrrolo[2,3-*d*]pyrimidines **19a**–**d** by thermolysis in diphenyl ether. ^1^H NMR showed no evidence of the presence of the other possible 6-substituted regioisomer (a key determinant is the presence of a C6-CH_3_ chemical shift). The aromatic esters of **19a**–**d** were subjected to base-catalyzed hydrolysis to afford pteroic acids **20a**–**d**. Compounds **20a**–**d** were subsequently coupled with l-glutamate diesters to afford **21a**–**d**. Final saponification of diesters **21a**–**d** with 1 N NaOH, followed by neutralization and acidification to pH 4 in the cold, provided target compounds **5**–**8**.

**Scheme 3:**
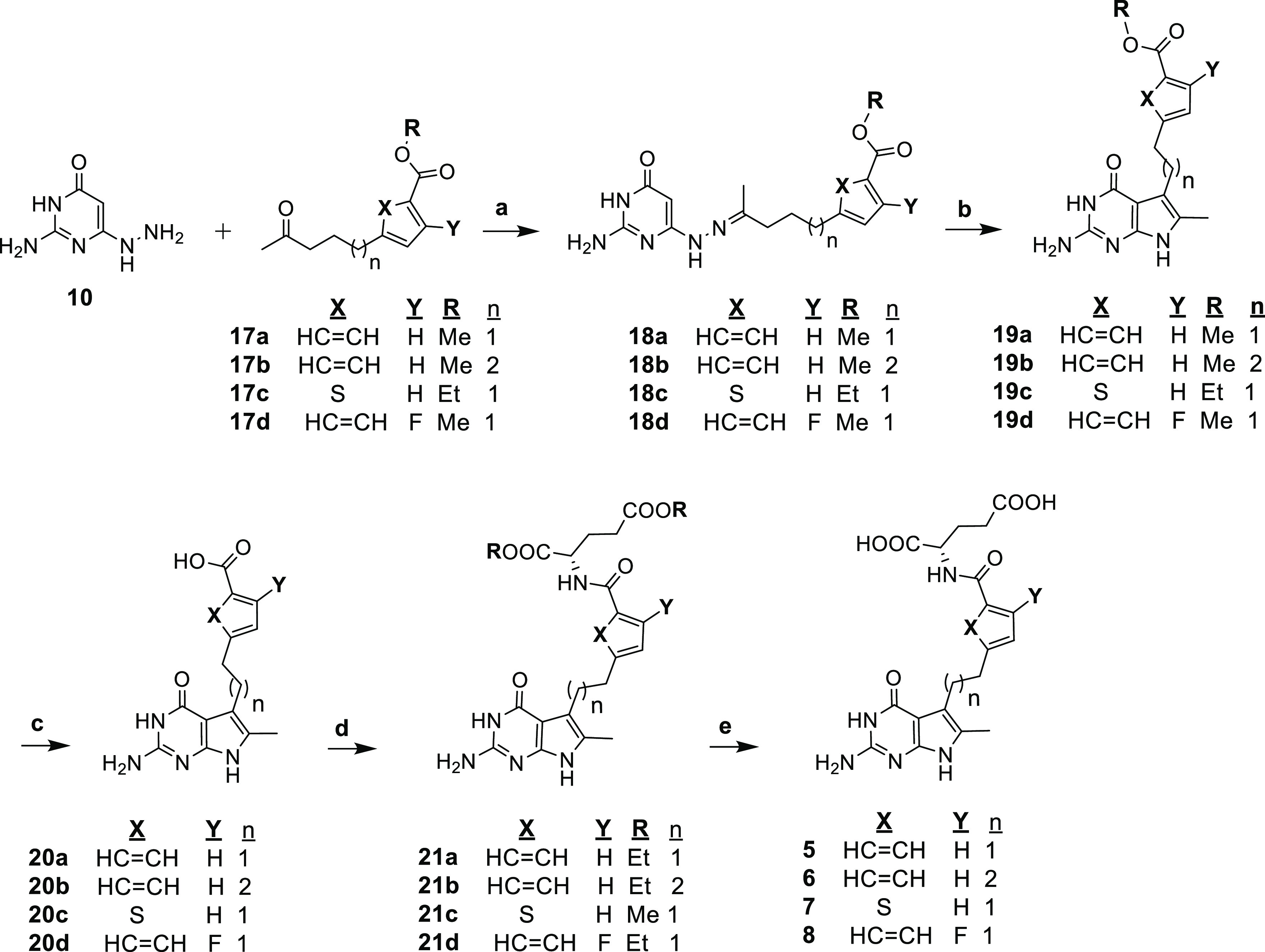
Reagents and conditions: (a) 2-methoxyethanol, reflux, 14 h, 56–89%; (b) Ph_2_O, 240 °C, or reflux, 5–8 h, 12–77%; (c) 1 N NaOH, rt, 12 h, 63–94%; (d) Dimethyl- or diethyl-l-glutamate, (i) NMM, CDMT, DMF, rt or 40 °C, 8–12 h, 30–78% or (ii) isobutyl chloroformate, TEA, DMF, 0 °C, to rt, 60 h, 61% ; (e) (i) 1 N NaOH, rt, 1–24 h; (ii) 0–4 °C, 1 N HCl, 19–80%.

For desmethyl compound **4**, a different synthetic strategy was used. A Sonogashira coupling reaction between bromofluorobenzoate **11d** and unsaturated alkynol **22** afforded **23** (Scheme 4), followed by hydrogenation and oxidation to obtain aldehyde **25**. Bromination afforded **26**, which was immediately condensed with 2,6-diamino-4-oxo-pyrimidine at 45 °C to afford the 5-substituted pyrrolo[2,3-*d*]pyrimidine **27**. Ester hydrolysis of **27**, followed by the amide coupling of **28** with l-glutamate diethyl ester hydrochloride, afforded **29**. The diester **29** was hydrolyzed with 1 N NaOH, followed by acidification to pH 4 at 0–4 °C, to provide compound **4**.

**Scheme 4:**
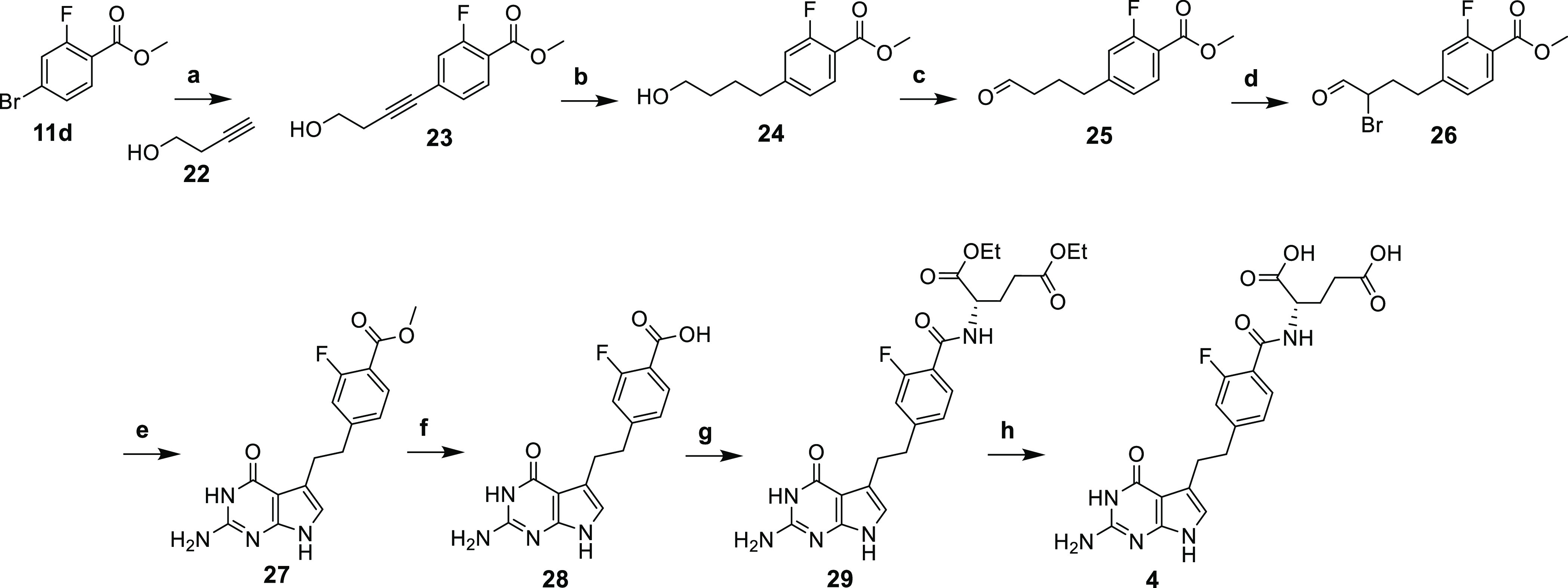
Reagents and conditions: (a) CuI, PdCl_2_, PPh_3_, Et_3_N, CH_3_CN, microwave, 100 °C, 1 h, 63%; (b) 10% Pd/C, H_2_, 55 psi, MeOH, rt, 9 h, 95%; (c) Dess-Martin Periodinane, CH_2_Cl_2_, 0 °C; to rt, 3 h, 45%, (d) Br_2_, 1,4-dioxane, CH_2_Cl_2_, rt, 3 h; (e) 2,6-diaminopyrimidin-4(3*H*)-one, CH_3_COONa, MeOH, 45 °C; 12 h, 46% over two steps; (f) 1 N NaOH, rt, 3 h, 96%; (g) *N*-methylmorpholine, 2-chloro-4,6-dimethoxy-1,3,5-triazine, l-glutamate diethyl ester hydrochloride, DMF, rt, 12 h, 59%;(h) (i) 1 N, NaOH, rt, 3 h; (ii) 0–4 °C, 1 N HCl, 56%.

To assess the impact of 6-methyl substitutions on the bicyclic scaffolds of the pyrrolo[2,3-*d*]pyrimidine antifolates **1**–**4** on RFC transport vis-à-vis PCFT, FRα, and FRβ, we used a unique biological readout for transport activity. This involved proliferation assays with isogenic Chinese hamster ovary (CHO) sublines derived from RFC-, FR-, and PCFT-null MTXRIIOua^R^2–4 CHO cells^39,40^ engineered to express the human FRα (RT16),^25^ FRβ (D4),^25^ RFC (PC43–10),^41^ or PCFT (R2/PCFT4).^26,42^ As these CHO cell lines are genetically identical except for their folate transporters, differences in inhibition of cell proliferation by specific cytotoxic antifolates directly reflect transporter specificities.^19,23−31,42^

RFC-expressing PC43–10 cells were potently inhibited by the 5-substituted pyrrolo[2,3-*d*]pyrimidine compounds **1**–**4** with half-maximal inhibitory concentration (IC_50_) values that ranged from 31 to 169 nM (Table 1). This extended to FRα-expressing RT16 (IC_50_ values of 6.8–550 nM), FRβ-expressing D4 (IC_50_ values of 3.5–552 nM), and PCFT-expressing R2/PCFT4 (22–329 nM) cells. We discovered that for the corresponding 6-methyl substituted pyrrolo[2,3-*d*]pyrimidine analogues **5**–**8**, RFC-mediated cell inhibition was uniformly abolished (IC_50_ > 1000 nM), with modest effects on FRα (RT16 IC_50_s from 2.5–100 nM) and FRβ (D4 IC_50_s from 2.6–49 nM) (Table 1). For PCFT-expressing R2/PCFT4 cells, 6-methyl pyrrole substitution completely preserved (**7**) or slightly increased (**5**, **6**) inhibition potencies (relative to compounds **3**, **1**, and **2**, respectively). However, the 6-methyl substitution in compound **8** abolished PCFT activity altogether. Transport specificities are further reflected in “Specificity Ratios”, defined as the ratios of IC_50_ values for PC43–10 (RFC) to those for R2/PCFT4 (PCFT), RT16 (FRα), or D4 (FRβ) cells (Table 1). Thus, our rationale to provide steric hindrance with a 6-methyl moiety to impede attachment to the RFC was indeed realized.

**Table 1:**
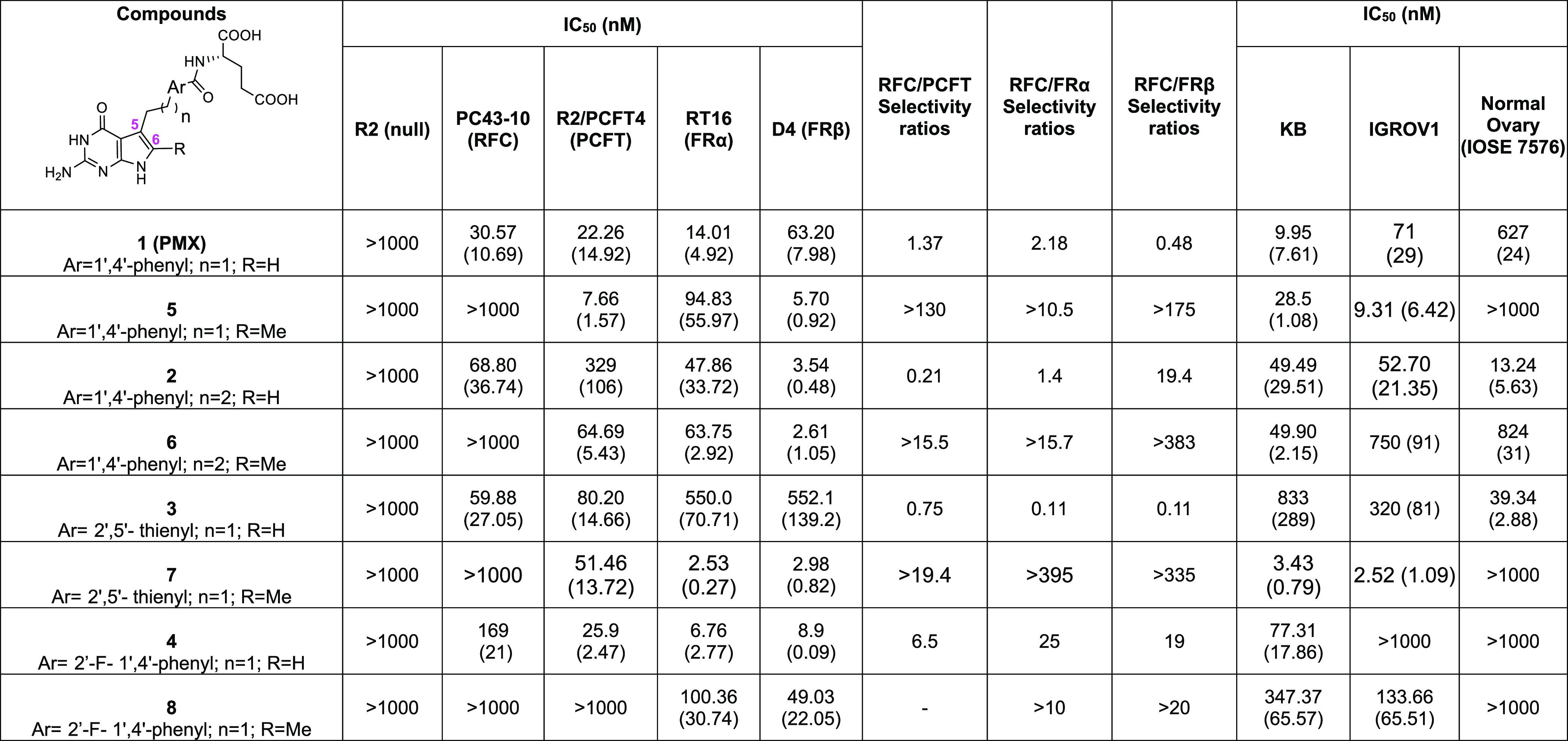
**Cell Proliferation (IC**_**50**_**nM) Assays with 6-Methyl 5-substituted Pyrrolo[2,3-***d***]pyrimidine Analogues**^a^

a Growth inhibition assays were performed using CHO sublines derived from RFC-, PCFT-, and FR-null MTXRIIOuaR2-4 CHO cells (R2)^39,40^ engineered to express human RFC (PC43-10),^41^ PCFT (R2/PCFT4),^26,42^ FRα (RT16),^25^ or FRβ (D4).^25^ Human cell lines include KB nasopharengeal carcinoma cells, IGROV1 ovarian cancer cells,^46^ and IOSE 7576 normal ovary cells.^43^ Experimental methods are described in the Supporting Information and were previously published.^23−26,28−31,35,42^ Results are shown as mean values from three to five experiments (± standard errors) and are presented as the calculated IC_50_ values representing the concentrations at which growth of 50% of cells was inhibited relative to untreated cells. “Selectivity Ratios” are ratios of IC_50_ values from the respective CHO sublines.

Table 1 also shows the inhibition of proliferation of KB human nasopharyngeal carcinoma cells and IGROV1 epithelial ovarian cancer cells, both of which express RFC, PCFT, and FRα.^27,42^ Results are compared to those with IOSE 7576 normal ovary cells^43^ which express RFC comparable to IGROV1 cells accompanying low levels of PCFT (∼30% of IGROV1) and undetectable FRα. For the tumor cells, except the des-methyl compound **4** with IGROV1 cells, all compounds were inhibitory. The 6-methyl substituted compounds (**5**, **6**, and **8**) inhibited KB cell proliferation with nanomolar IC_50_ values only slightly less than those for the nonmethylated 5-substituted (**1**, **2**, and **4**) pyrrolo[2,3-*d*]pyrimidine compounds. For both KB and IGROV1 cells, compound **7** was the most potent inhibitor with 242-fold and 127-fold, respectively, greater potencies than the corresponding 6-desmethyl analogue **3**.

Des-methyl compounds **2** and **3** were more potent toward IOSE 7576 cells than IGROV1 cells (Table 1). Whereas compound **1** (PMX) was more active toward IGROV1 cells, **4** was inactive toward both IGROV1 and IOSE 7576 cells. Notably, the 6-methyl-substituted compounds **5**, **7**, and **8** were far more potent than the corresponding des-methyl compounds (**1**, **3**, and **4**) toward IGROV1 cells. Conversely, compounds **5**, **7**, and **8** were inert toward IOSE 7576 cells up to 1000 nM. Compound **6** was modestly inhibitory toward both IGROV1 and IOSE 7576 cells. Thus, 6-methyl substitution increases potency toward IGROV1 ovarian cancer cells over IOSE 7576 normal ovary cells.

To identify the targeted pathways/enzymes of these pyrrolo[2,3-*d*]pyrimidine compounds (**1**, **2**, **4**–**8**), *in vitro* growth inhibition assays were performed with KB tumor cells in glycine-, adenosine-, and thymidine-free media without additions or in the presence of thymidine (10 μM), adenosine (60 μM), or glycine (130 μM) (both singly and in various combinations; Figure 4).^18,23−25,28−31,42,44,45^ For *de novo* purine biosynthesis inhibitors, protection was also tested with AICA (320 μM), which is metabolized to AICA ribonucleotide (substrate for the AICARFTase reaction, thus circumventing GARFTase).

**Figure 4:**
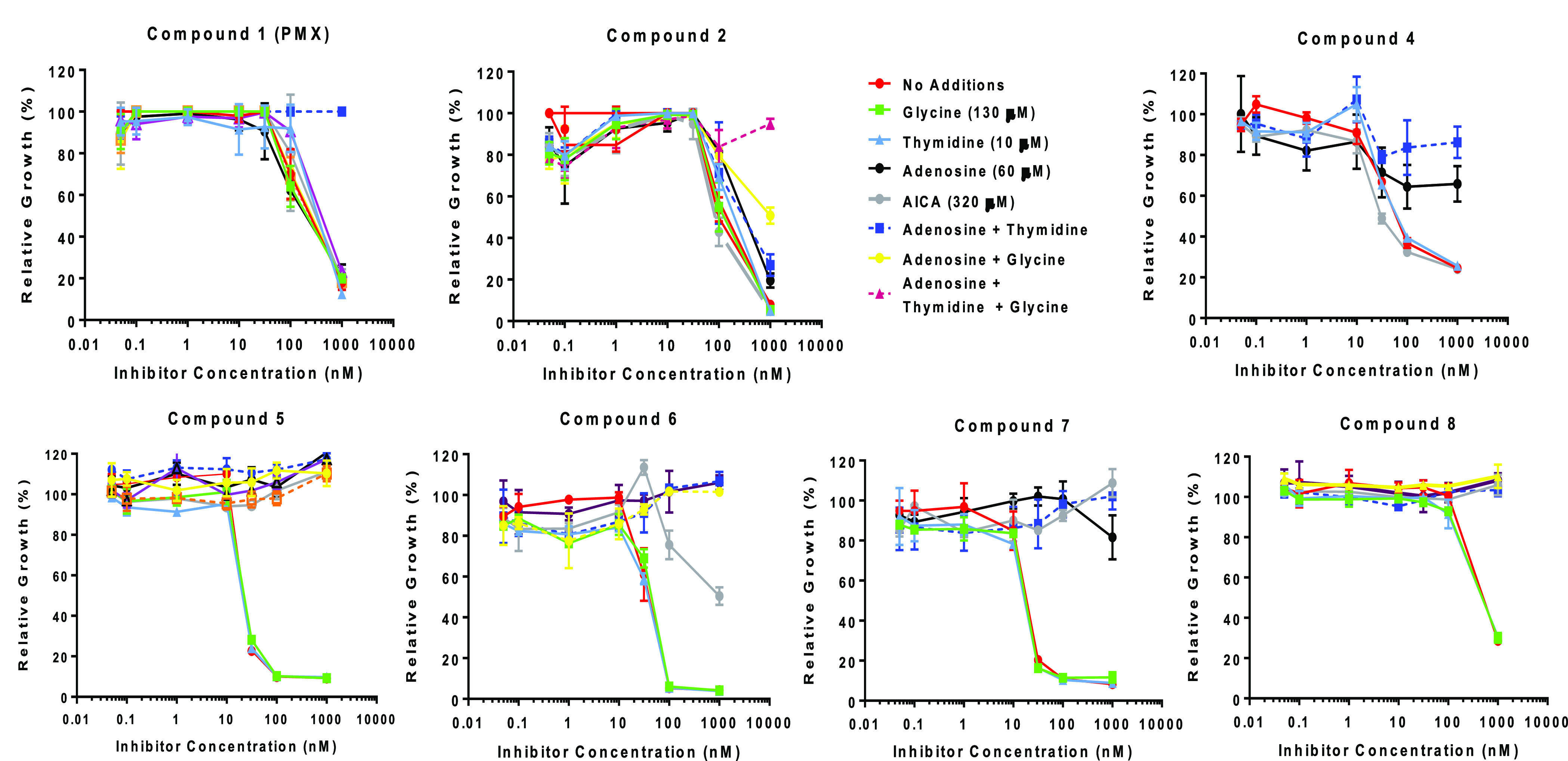
**Metabolite protection experiments.** To demonstrate the intracellular target(s) for the pyrrolo[2,3-*d*]pyrimidine antifolates (**1**, **2**, **4**–**8**), proliferation of KB cells was assayed in the presence of the inhibitors with nucleosides (adenosine, thymidine), glycine, and 5-aminoimidazole-4-carboxamide (AICA). Cell densities were measured with a fluorescence assay^25^ and a fluorescence plate reader. Results were normalized to cell densities in the absence of drug. Results shown are mean values plus/minus standard errors from triplicate experiments. Experimental details are described in the Supporting Information, and the methods have been previously published.^18,23−25,28−31,42,44^

The 5-subsituted pyrrolo[2,3-*d*]pyrimidine compounds showed patterns of metabolite protection consistent with the inhibition of multiple C1 pathways. Interestingly, compounds **2** and **4** like PMX (**1**) consistently inhibited both thymidylate and purine nucleotide biosynthesis (protected by adenosine and thymidine), the latter most likely at AICARFTase as well as at GARFTase (protection by AICA).^23−25,28−31,35,42^ For compound **2**, addition of glycine provided greater protection than did adenosine and thymidine alone. This suggests an inhibition of mitochondrial C1 metabolism in addition to nucleotide biosynthesis in the cytosol.^45^ Conversely, the 6-methyl-substituted compounds **5**–**8** were exclusive inhibitors of *de novo* purine biosynthesis (protected by adenosine alone), most likely at GARFTase (protected by AICA).^23−25,28−31,35,42^

In summary, a methyl group plays a vital role in the molecular recognition of endogenous and exogenous ligands by receptors.^47^ Further, the steric and electronic effects of a methyl group can influence selective ligand binding and increased potency, among various other pharmacologic effects.^48−50^

In the current study, we synthesized 6-methyl 5-substituted pyrrolo[2,3-*d*]pyrimidine compounds (**5**, **6**, **7**, and **8**) as tumor-targeted antifolates based on the structures of the nonmethylated 5-substituted pyrrolo[2,3-*d*]pyrimidine compounds **1**, **2**, **3**, and **4**.^23,24,3^ Our goal was to explore the impact of 6-methyl substitutions on drug uptake and cell inhibition mediated by RFC vis á vis more tumor-selective PCFT, FRα, and FRβ.^3,4,6,8,13,21^ A compelling rationale for our study was provided from molecular modeling of our pyrrolo[2,3-*d*]pyrimidine antifolates in the RFC structure^37^ which indicated a steric clash of the 6-methyl moiety of compounds **5**–**8** with Tyr126 in the RFC binding site; no analogous feature was seen for PCFT or for FRs α or β. As a biological readout of transport, we used our isogenic panel of CHO sublines.^25,41,42^

Consistent with our prior studies,^23,24^ we confirmed that 5-substituted pyrrolo[2,3-*d*]pyrimidine antifolates **1**–**4** without 6-methyl substitutions exhibited promiscuous transport by RFC as well as by PCFT and FRs α and β. In KB cells, the loss of cell proliferation reflected inhibition of thymidylate biosynthesis and purine nucleotide biosynthesis at both GARFTase and AICARFTase. For compound **2**, inhibition of mitochondrial C1 metabolism was also suggested. We discovered that 6-methyl-substituted pyrrolo[2,3-*d*]pyrimidine antifolates **5**–**8** are distinctly selective for transport by PCFT and FRs over RFC and exclusively target *de novo* purine biosynthesis at GARFTase.^25,28−31^ Selectivity ratios for the 6-methyl compounds with PCFT ranged from >15.5 (**6**) to >130 (**5**), whereas for FRα selectivity ranged from >10 (**8**) to >395 (**7**). For FRβ, selectivity ratios ranged from >20 (**8**) to >383 (**6**). Inhibition of cell proliferation by the 6-methyl-substituted pyrrolo[2,3-*d*]pyrimidine antifolates **5**–**8** was extended to IGROV1 cells with comparison to IOSE 7576 cells, demonstrating that the 6-methyl substitution promotes tumor selectivity.

In conclusion, we established that 6-methyl substitutions on the pyrrole ring of 5-substituted pyrrolo[2,3-*d*]pyrimidine analogues preserved or increased inhibitor potencies and tumor selectivity, while positively impacting drug uptake by FRs and PCFT over RFC. Improved target selectivity, potency, and modified mechanisms-of-action (including multienzyme inhibition) provide compelling evidence of the profound impact of minor structural alterations in antitumor drug polypharmacology for tumor-targeted pyrrolo[2,3-*d*]pyrimidine antifolates. Clearly, the 6-methyl group provides an elegantly simple structural solution that would afford significant advantages over current clinically used antifolates.

## Supporting Information Available

Additional molecular modeling figures, synthetic experimental details, and biological assay methods (PDF)
