# Supplementary material for: Discovery of Tumor-Targeted 6-Methyl Substituted Pemetrexed and Related Antifolates with Selective Loss of RFC Transport
Source: ACS Med Chem Lett. 2023 Nov 15;14(12):1682–91. doi: 10.1021/acsmedchemlett.3c00326 (PMC10726441; doi:10.1021/acsmedchemlett.3c00326)
Supplement: Supplementary file 1 — ml3c00326_si_001.pdf [file ml3c00326_si_001.pdf]

SUPPORTING INFORMATION

Discovery of Tumor-Targeted 6-Methyl Substituted Pemetrexed and Related Antifolates With Selective Loss of RFC Transport

Krishna Kaku[£], Manasa P. Ravindra[£], Nian Tong[£], Shruti Choudhary[£], Xinxin Li[£], Jianming Yu[£], Mohammad Karim[£], Madelyn Brzezinski[‡], Carrie O'Connor[‡], Zhanjun Hou[||‡], Larry H. Matherly[||‡§θ*], and Aleem Gangjee[£θ*]

[£]Division of Medicinal Chemistry, Graduate School of Pharmaceutical Sciences, Duquesne University, 600 Forbes Avenue, Pittsburgh, Pennsylvania 15282 412-396-6070; gangjee@duq.edu.

[||]Molecular Therapeutics Program, Barbara Ann Karmanos Cancer Institute, 4100 John R, Detroit, Michigan 48201 313-578-4280; matherly@karmanos.org

[‡]Department of Oncology, Wayne State University School of Medicine, Detroit, Michigan 48201, United States

[§]Department of Pharmacology, Wayne State University School of Medicine, Detroit, Michigan 48201, United States

[θ]These authors contributed equally to this work.

*To whom correspondence should be addressed.

Table of Contents

I. Molecular Modeling of Compounds **6**-**8** in RFC S3

II. Synthetic Procedures S3

III. Molecular Modeling Protocol S16

IV. Biological Assay Protocols S16

I. Molecular Modeling of Compounds 6-8 in RFC:

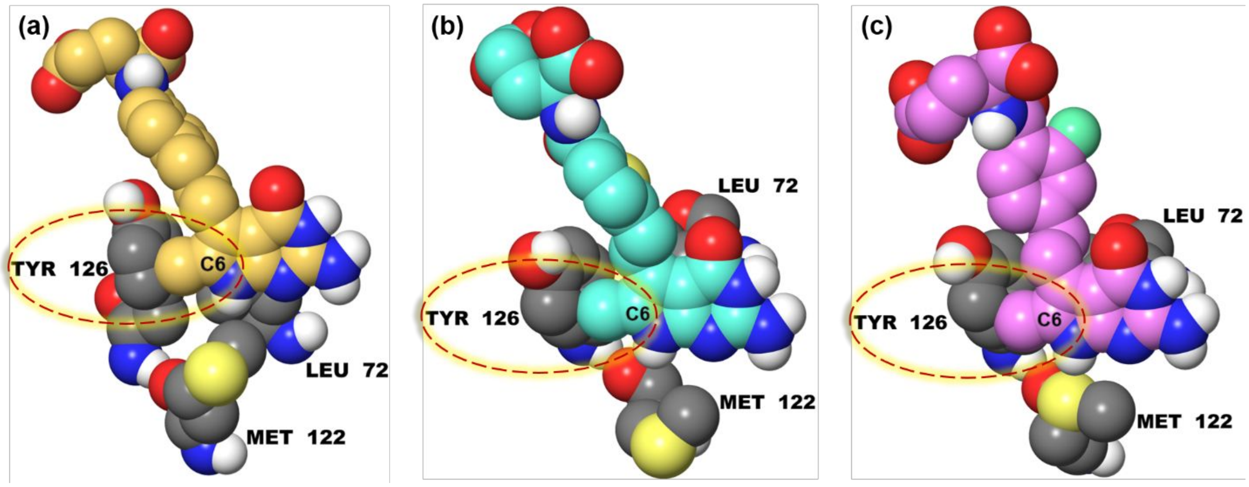

Fig. S1: Docked poses of (a) **6** (yellow), (b) **7** (cyan) and (c) **8** (pink) in the binding pocket of the reduced folate carrier (SLC19A1; RFC) (PDB: 8GOF), as space-filled models. The steric clash with Tyr126 is highlighted.

II. Experimental section

Synthetic Procedures

All evaporations were carried out in vacuum with a rotary evaporator. Analytical samples were dried in vacuo in a CHEM-DRY drying apparatus over P_2O_5 at 50 ˚C. Melting points were determined either using a MEL-TEMP II melting point apparatus with FLUKE 51 K/J electronic thermometer or using an MPA100 OptiMelt automated melting point system and are uncorrected. Nuclear magnetic resonance spectra for proton (^{1}H NMR) were recorded on the Bruker WH-300 (300 MHz) or Bruker Avance II 400 (400 MHz) or Bruker Avance II 500 (500 MHz) NMR systems with TopSpin processing software. ^{1}H spectra were referenced to TMS and trifluoroacetic acid, respectively, as the internal standards to express the chemical shift values (δ) in ppm (parts per million): s, singlet; d, doublet; dd, doublet of doublet; t, triplet; q, quartet; m, multiplet; br, broad singlet; td, triplet of doublet; dt, doublet of triplet; quin, quintet. ^{1}H NMR of alkyl CH_2s of target compounds merged with DMSO and water peaks have been omitted from the experimental whenever not visible in ^{1}H NMR. ^{1}H NMR of alkyl CH_2s of intermediate compounds within DMSO and water peaks and D_2O exchangeable peaks that were exchanged due to excess moisture have also been omitted from the experimental whenever not visible in ^{1}H NMR. Thin-layer chromatography (TLC) was performed on Whatman® PE SIL G/UV254 flexible silica gel plates and the spots were visualized under 254 and 365 nm ultraviolet illumination. Proportions of solvents used for TLC are by volume. All analytical samples were homogeneous on TLC in at least two different solvent systems. Column chromatography was performed on the silica gel (70 to 230 meshes, Fisher Scientific) column. Flash chromatography was carried out on the CombiFlash® *Rf* systems, model COMBIFLASH *RF*. Pre-packed RediSep® *Rf* normal-phase flash columns (230 to 400 meshes) of various sizes were used. The amount (weight) of silica gel/celite for column

chromatography was in the range of 5-10 times the amount (weight) of the crude reaction mixture being separated. Elemental analyses were performed by Atlantic Microlab, Inc., Norcross, GA. Element compositions are within ± 0.4% of the calculated values. Fractional moles of water or organic solvents frequently found in some analytical samples could not be prevented despite 24 to 48 hours of drying in vacuo and were confirmed where possible by their presence in the ^{1}H NMR spectra. High Performance Liquid Chromatography (HPLC) was performed on Waters® 600E Multisolvent Delivery System, components: Waters® 600 Controller (model code: 600), 600E Pump (model code: 60F), Waters® 2487 Dual λ Absorbance Detector and Vanquish Core HPLC System (ThermoFisher Scientific), components: Multiwavelength detector CG (model code: VC-D12-A-01), Split sampler C (model code: VC-A13-A-02), Quaternary pump C (model code: VC-P20-A-01), Column compartment C (model code: VC-C10-A-03), Fraction Collector F (model code: VF-F11-A-01). Mobile phase was an aqueous blend of 0.1% formic acid in water with a miscible, polar organic solvent acetonitrile. High-resolution Mass Spectrometry (HRMS) data were acquired using the ESI probe on a Thermo Scientific LTQ Orbitrap XL system.

2-Amino-6-hydrazineylpyrimidin-4(3H)-one (10)

To a stirred suspension of **9** (2 g, 13.74 mmol) in water (20 mL) was added hydrazine hydrate (2.5 g, 50 mmol), and the mixture was heated at reflux for 3 h. The resulting clear solution was cooled, and the precipitate that separated was collected by filtration, washed with water followed by ethanol and dried to give **10** (1.4 g, 72%) as a buff colored powder. mp 274 ˚C dec. Compound **10** spots very faintly and doesn't run on TLC ($CHCl_3$/MeOH). ^{1}H NMR (400 MHz, DMSO-d_6): δ 4.03 (s, 2 H, NH-$\underline{NH_2}$, exch), 4.77 (s, 1 H, 6-NH, exch), 6.10 (s, 2 H, 2-NH_2, exch), 7.25 (s, 1 H, C5-CH), 9.65 (s, 1 H, 3-NH, exch). ^{1}H NMR matches with the reported NMR.[1]

Methyl 4-(5-oxohex-1-en-1-yl)benzoate (14)

To a 150 mL round bottom flask were added methyl 4-iodobenzoate **11a** (**Z** = I) (1.0 g, 4.65 mmol), $Pd(OAc)_2$ (0.05 g, 0.23 mmol), hex-5-en-2-one **13** (0.91 g, 9.3 mmol), Bu_4NCl (1.29 g, 4.7 mmol), KOAc (1.37 g, 13.9 mmol), and DMF (70 mL). The mixture was stirred at 80 ˚C overnight. After cooling to room temperature, the mixture was diluted with saturated $NaHCO_3$ solution (80 mL) and extracted with ethyl ether (2 x 100 mL). The ether extracts were dried over anhydrous Na_2SO_4, concentrated under reduced pressure, and the residue was loaded onto a silica gel column and eluted with 4:1 hexanes/ethyl acetate to give **14** (1.05 g, 97%) as a yellow solid. TLC R_f 0.60 (ethyl acetate/hexanes, 1:1); mp 63-65 ˚C; ^{1}H NMR (300 MHz, $CDCl_3$) δ 2.18 (s, 3 H, $COCH_3$), 2.52 (q, 2 H, J = 6.6 Hz, CH_2), 2.64 (t, 2 H, J = 6.6 Hz, CH_2), 3. 90 (s, 3 H, OCH_3), 6.33 (dt, 1 H, J = 15.8 Hz, J = 6.3 Hz, CH), 6.45 (d, 1 H, J = 15.8 Hz, CH), 7.37 (d, 2 H, J = 8.6 Hz, Ar), 7.95 (d, 2 H, J = 8.6 Hz, Ar).

Methyl 2-fluoro-4-(4-hydroxypent-1-en-1-yl)benzoate (15)

To a round bottom flask were added methyl 4-bromo-2-fluorobenzoate **11d** (**Z** = Br) (4.0 g, 17.16 mmol) and DMF (100 mL), followed by pent-4-en-2-ol **12** (1.94 mL, 18.88 mmol), TEA (2.63 mL, 18.88 mmol), $Pd(dba)_2$ (786 mg, 0.86 mmol) and 2-(di-tert-butylphosphino)-1-phenylindole (0.35 g, 1.03 mmol). The reaction was stirred at 100 ˚C

for 2h. After cooling to room temperature, ethyl acetate (100 mL) was added and the mixture was washed with H_2O (3 x 150 mL), dried over anhydrous Na_2SO_4, concentrated under reduced pressure. The crude product was dissolved in MeOH. To the solution, silica gel was added and the solvent was evaporated to afford a plug. The plug was loaded on a silica gel column and flash-chromatographed with hexanes/ethyl acetate gradient elution. The fractions with desired Rf were pooled, evaporated and the residue was dried in vacuo using P_2O_5 to afford **15** (2.13 g, 8.94 mmol) as an oily liquid in 52% yield. TLC R_f 0.51 (ethyl acetate/hexanes, 1:1); ^{1}H NMR (400 MHz, DMSO-d_6): δ 1.09-1.11 (d, 3 H, CH_3), 2.23-2.35 (m, 2 H, CH_2), 3.73-3.78 (m, 1 H, CH), 3.84 (s, 3 H, OCH_3), 4.63-4.64 (d, 1 H, O<u>*H*</u>, exch), 6.45-6.61 (m, 2 H, CH), 7.34-7.41 (m, 2H, Ar), 7.81-7.85 (t, 1H, Ar).

General procedure A for the synthesis of 17a, 17c

To a solution of halogenated aromatic ester **11a** or **11c** (1eq) in DMF (20mL) was added alkenol **12**, LiCl, LiOAc, Bu_4NCl, $Pd(OAc)_2$ and the mixture was stirred at 80°C for 3 to 16 hours. TLC showed the disappearance of the starting material and formation of one major polar spot (hexanes/ ethyl acetate). To the reaction mixture cooled to room temperature was added ethyl acetate. The resulting solution was extracted with H_2O and dried over Na_2SO_4. After evaporation of solvent, a silica gel plug of the residue was loaded on a silica gel column and flash-chromatographed with hexane followed by gradual increase in polarity to 100% ethyl acetate. The desired fractions were pooled, evaporated and the residue was dried in vacuo using P_2O_5 to afford **17a** and **17c**.

Methyl 4-(4-oxopentyl)benzoate (17a)

Compound **17a** was synthesized from methyl 4-iodobenzoate **11a** (**Z** = I) (1.4 g, 5 mmol) in DMF (20 mL), pent-4-en-2-ol **12** (0.52 ml, 5 mmol), LiCl (212 mg, 5 mmol), LiOAc (825 mg, 12.5 mmol), Bu_4NCl (1.41 g, 5 mmol), and $Pd(OAc)_2$ (68 mg, 0.3 mmol) according to general procedure A, to afford **17a** (788 mg, 72%) as a colorless syrup. TLC R_f = 0.42 (hexanes/ethyl acetate, 3:1); ^{1}H NMR (400 MHz, DMSO-d_6): δ 1.73-1.81 (m, 2 H, CH_2), 2.07 (s, 3 H, $COCH_3$), 2.42-2.46 (t, J = 7.31, 7.31 Hz, 2 H, Ar-CH_2), 2.6-2.64 (m, 2 H, CH_2), 3.84 (s, 3 H, $COOCH_3$), 7.33-7.36 (d, J = 8.33 Hz, 2 H, Ar), 7.87-7.89 (d, J = 8.30 Hz, 2 H). ^{1}H NMR matches with the reported NMR.[1]

Methyl 4-(5-oxohexyl)benzoate (17b).

To a Parr hydrogenation bottle was added **14** (1.0 g, 4.31 mmol) dissolved in MeOH (50 mL), followed by the addition of 5% Pd/C (0.20 g). The resulting mixture was hydrogenated at 35 psi for 24 h. After filtering and washing the catalyst thoroughly with methanol, the filtrate was concentrated in vacuo to afford of **17b** (0.90 g, 90%) as a colorless oil. TLC R_f 0.68 (ethyl acetate/hexanes, 1:1); ^{1}H NMR (300 MHz, $CDCl_3$) δ 1.61 (m, 4 H, CH_2CH_2), 2.11 (s, 3 H, CH_3), 2.44 (t, 2 H, J = 6.9 Hz, CH_2), 2.66 (t, 2 H, J = 6.9 Hz, CH_2), 3.89 (s, 3 H, CH_3), 7.23 (d, 2 H, J = 8.2 Hz, Ar), 7.94 (d, 2 H, J = 8.2 Hz, Ar). ^{1}H NMR matches with the reported NMR.[2]

Ethyl 5-(4-oxopentyl)thiophene-2-carboxylate (17c)

Compound **17c** was synthesized from ethyl 5-bromothiophene-2-carboxylate **11c** (**Z** = Br) (10 g, 42.54 mmol) in DMF (100 mL), pent-4-en-2-ol **12** (4.4 ml, 42.54 mmol), LiCl (1.8 g, 42.54 mmol), LiOAc (8.42 g, 127.61 mmol), Bu_4NCl (11.82 g, 42.54 mmol), and $Pd(OAc)_2$

(477.5 mg, 2.13 mmol) according to general procedure A, to afford **17c** (7.15 g, 70%) as an orange colored oil. TLC R_f = 0.25 (hexanes/ethyl acetate, 2:1); ^{1}H NMR (400 MHz, DMSO-d_6) δ 1.28 (t, J = 7.1 Hz, 3 H, $COOCH_2\underline{CH_3}$), 1.85-1.76 (m, 2 H CH_2), 2.08 (d, J = 0.5 Hz, 3 H, $COCH_3$), 2.48 (d, J = 7.2 Hz, 2 H, CH_2), 2.81 (ddd, J = 7.9, 7.1, 0.9 Hz, 2 H, CH_2), 4.26 (q, J = 7.1 Hz, 2 H, $COO\underline{CH_2}CH_3$), 6.96 (dt, J = 3.8, 0.9 Hz, 1 H, Ar), 7.64 (d, J = 3.7 Hz, 1 H, Ar). ^{13}C NMR (125 MHz, DMSO-d_6) δ 208.41, 161.83, 153.48, 134.23, 130.92, 126.61, 61.17, 42.08, 30.18, 29.32, 25.40, 14.64.

Methyl 2-fluoro-4-(4-oxopentyl)benzoate (17d)
To a Parr hydrogenation bottle was added **15** (2.13 g, 8.93 mmol) dissolved in MeOH (50 mL), followed by the addition of 5% Pd/C (0.20 g). The resulting mixture was hydrogenated at 35 psi for 24 h. After filtering and washing the catalyst thoroughly with methanol, the filtrate was concentrated in vacuo to afford of **16** (1.82 g, 7.6 mmol) as a colorless oil in 89.5 % yield. TLC R_f 0.43 (hexanes/ethyl acetate, 1:1); ^{1}H NMR (400 MHz, DMSO-d_6) δ 1.02-1.04 (d, 3 H, CH_3), 1.28-1.35 (m, 2 H, CH_2), 1.53-1.71 (m, 2 H, CH_2), 2.62-2.67 (m, 2 H, CH_2), 3.55-3.62 (m, 1 H, CH), 3.84 (s, 3 H, OCH_3), 4.38-4.39 (d, 1 H, O*H*, exch,), 7.16-7.22 (m, 2 H, Ar), 7.79-7.83 (t, 1 H, Ar). Compound **16** was utilized in the next reaction without further purification. Compound **16** (1.82 g, 7.6 mmol) was dissolved in $CHCl_3$ at room temperature after which DMP reagent was added (4.5 g, 10.6 mmol) and stirred for 3 hours. The compound was extracted with $CHCl_3$ after a basic workup to yield methyl 2-fluoro-4-(4-oxopentyl)benzoate **17d** as an oily liquid in 67% yield (1.21 g, 5.09 mmol). TLC R_f 0.59 (hexanes/ethyl acetate, 1:1); ^{1}H NMR (400 MHz, DMSO-d_6) δ 1.74-1.81 (m, 2 H, CH_2), 2.07 (s, 3 H, CH_3), 2.42-2.46 (t, 2 H, CH_2), 2.60-2.64 (t, 2 H, CH_2), 3.84 (s, 3 H, OCH_3), 7.16-7.22 (m, 2 H, Ar), 7.79-7.83 (t, 1 H, Ar). ^{13}C NMR (125 MHz, Chloroform-*d*) δ 208.24, 164.93, 163.08, 149.67, 132.21, 124.17, 116.87, 116.16, 52.27, 42.46, 34.71, 30.08, 24.37.

Methyl-4-(4-(2-(2-amino-6-oxo-1,6-dihydropyrimidin-4-yl)hydrazineylidene)pentyl)benzoate (18a)
A mixture of the ketone **17a** (591 mg, 2.52 mmol) and 2-amino-6-hydrazineylpyrimidin-4(3*H*)-one **10** (356 mg, 2.52 mmol) in 2-methoxyethanol (20 mL) was refluxed for 14 h, then cooled to room temperature and filtered. The filtrate was concentrated, to which 1:1 ether/hexanes were added, and the resulting brownish powder was collected by filtration to afford **18a** (770 mg, 89%) as a light brown powder. TLC R_f 0.2 ($CHCl_3$/MeOH, 5:1); mp 240 ˚C.[1] ^{1}H NMR (400 MHz, DMSO-d_6): δ 1.78-86 (m, 5 H, $CNCH_3$, CH_2), 2.2-2.24 (t, J=7.35, 7.35 Hz, 2 H, CH_2), 2.66-2.7 (t, J=7.58, 7.58 Hz, 2 H, CH_2), 3.84 (s, 3 H, $COOCH_3$), 5.09 (s, 1 H, C5-CH), 6.18 (s, 2 H, 2-NH_2, exch), 7.36-7.42 (m, 2 H, Ar), 7.88-7.89 (d, J = 8.22 Hz, 2 H, Ar), 8.75 (s, 1 H, 6-NH, exch), 9.95 (s, 1 H, 3-NH, exch). ^{1}H NMR matches with the reported NMR.[1]

Methyl-4-(5-(2-(2-amino-6-oxo-1,6-dihydropyrimidin-4-yl)hydrazineylidene)hexyl)benzoate (18b)
A mixture of the ketone **17b** (1 g, 4.27 mmol) and 2-amino-6-hydrazineylpyrimidin-4(3*H*)-one **10** (0.67 g, 4.27 mmol) in 2-methoxyethanol (20 mL) was refluxed for 14 h, then cooled to room temperature and filtered. The resulting solid was suspended in MeOH (50 mL) and silica gel (10g) was added and the solvent was evaporated to afford a plug. The

silica gel plug obtained was loaded onto a silica gel column and eluted with a gradient of 5-10% MeOH in $CHCl_3$ to afford **18b** (1 g, 72%) as a pale-yellow solid. TLC R_f 0.37 ($CHCl_3$/MeOH, 5:1); mp 236-237.5 ˚C. ^{1}H NMR (300 MHz, DMSO-d_6): δ 1.44-1.60 (m, 4 H, CH_2CH_2), 1.86 (s, 3 H, CH_3), 2.23 (t, 2 H, J = 7.2 Hz, CH_2), 2.68 (t, 2 H, J = 7.2 Hz, CH_2), 3.83 (s, 3 H, CH_3), 5.07 (s, 1 H, C5-CH), 6.08 (s, 2 H, 2-NH_2, exch), 7.36 (d, 2 H, J = 8.2 Hz, Ar), 7.87 (d, 2 H, J = 8.2 Hz, Ar), 8.68 (s, 1 H, 6-NH, exch), 9.89 (s, 1 H, 3-NH, exch). Anal. Calcd. For: $C_{18}H_{23}N_5O_3$: C, 60.49; H, 6.49; N, 19.59. Found: C, 60.23; H, 6.44; N, 19.77.

Ethyl-5-(4-(2-(2-amino-6-oxo-1,6-dihydropyrimidin-4-yl)hydrazineylidene)pentyl)thiophene-2-carboxylate (18c)

A mixture of the ketone **17c** (1.1 g, 4.6 mmol) and 2-amino-6-hydrazineylpyrimidin-4(3*H*)-one **10** (650 mg, 4.6 mmol) in 2-methoxyethanol (25 mL) was refluxed for 14 h, then cooled to room temperature and filtered. The filtrate was concentrated, to which 1:1 ether/hexanes were added, and the resulting precipitate was collected by filtration to afford **18c** (945 mg, 56%) as an off-white powder. TLC R_f 0.64 ($CHCl_3$/MeOH, 5:1 with 2 drops of 25% NH_4OH); mp 219.2-221 ˚C. ^{1}H NMR (500 MHz, DMSO-d_6) δ 1.28 (td, J = 7.1, 1.4 Hz, 3 H, $COOCH_2\underline{CH_3}$), 1.91-1.83 (m, 5 H, $CNCH_3$, CH_2), 2.27 (t, J = 7.3 Hz, 2 H, CH_2), 2.87 (t, J = 7.6 Hz, 2 H, CH_2), 4.26 (qd, J = 7.1, 1.0 Hz, 2 H, $COO\underline{CH_2}CH_3$), 5.08 (d, J = 6.3 Hz, 1 H, C5-CH), 6.15 (d, J = 18.3 Hz, 2 H, 2-NH_2, exch), 6.98 (dd, J = 3.8, 0.9 Hz, 1 H, Ar), 7.64 (d, J = 3.7 Hz, 1 H, Ar), 8.75 (s, 1 H, 6-NH, exch), 9.94 (s, 1 H, 3-NH, exch).

Methyl-4-(4-(2-(2-amino-6-oxo-1,6-dihydropyrimidin-4-yl)hydrazineylidene)pentyl)-2-fluorobenzoate (18d)

A mixture of the ketone **17d** (1.21 g, 5.09 mmol) and 2-amino-6-hydrazineylpyrimidin-4(3*H*)-one **10** (0.716 g, 5.08 mmol) in 2-methoxyethanol (20 mL) was refluxed for 14 h, then cooled to room temperature and filtered. The filtrate was concentrated, to which 1:1 ether/hexanes were added, and the resulting suspension was filtered to afford **18d** (1.41 g, 3.90 mmol) as a light brown powder in 77% yield. TLC R_f 0.32 ($CHCl_3$/MeOH, 10:1 with 2 drops of 25% NH_4OH); mp 253-255.3 ˚C; ^{1}H NMR (DMSO-d_6) δ 1.81-1.90 (m, 5 H, CH_2, CH_3), 2.20-2.24 (t, 2 H, CH_2), 2.65-2.69 (t, 2 H, CH_2), 3.84 (s, 3 H, OCH_3), 5.08 (s, 1 H, Ar), 6.18 (s, 2 H, NH_2, exch.), 7.18-7.25 (dd, 2 H, Ar), 7.79-7.83 (t, 1 H, Ar), 8.76 (s, 1 H, NH, exch.), 9.95 (s, 1 H, NH, exch.).

Methyl 4-(2-(2-amino-6-methyl-4-oxo-4,7-dihydro-3*H*-pyrrolo[2,3-*d*]pyrimidin-5-yl)ethyl)benzoate (19a)

A mixture of **18a** (350 mg, 1.02 mmol), in diphenyl ether (20 mL) was stirred and heated at reflux for 6 h. After cooling to room temperature, hexanes were added and the precipitated powder was collected by filtration and run through a silica column with 5% MeOH in $CHCl_3$ as the eluent to give cyclized **19a** (255 mg, 77%) as a yellow powder. TLC R_f 0.25 ($CHCl_3$/MeOH, 5:1); mp 260 ˚C.[1] ^{1}H NMR (400 MHz, DMSO-d_6): δ 1.83 (s, 3 H, Ar-CH_3), 2.75-2.79 (t, J = 7.29, 7.29 Hz, 2 H, Ar-CH_2), 2.89-2.93 (m, 2 H, Ar-CH_2), 3.83 (s, 3 H, $COOCH_3$), 5.96 (s, 2 H, 2-NH_2, exch), 7.26-7.28 (d, J = 8.20 Hz, 2 H, Ar), 7.83-7.85 (d, J = 8.16 Hz, 2 H, Ar), 10.1 (s, 1 H, 3-NH, exch), 10.5 (s, 1 H, 7-NH, exch). ^{1}H NMR matches with the reported NMR.[1]

Methyl 4-(3-(2-amino-6-methyl-4-oxo-4,7-dihydro-3*H*-pyrrolo[2,3-*d*]pyrimidin-5-yl)propyl)benzoate (19b)

A mixture of **18b** (0.90 g, 2.73 mmol) in diphenyl ether (25 mL) was stirred under N_2 and heated at reflux for 5 h. After cooling to room temperature, hexanes (100 mL) was added and the precipitated solid was collected by filtration. The resulting solid was suspended in MeOH (50 mL) and silica gel (10 g) was added and the solvent was evaporated to afford a plug. The silica gel plug obtained was loaded onto a silica gel column and eluted with a gradient of 5-10% MeOH in $CHCl_3$ to obtain **19b** (0.47 g, 57%) as a pale-yellow solid. TLC R_f 0.44 ($CHCl_3$/MeOH, 5:1); mp 230 ˚C dec. ^{1}H NMR (300 MHz, DMSO-d_6): δ 1.82 (m, 2 H, CH_2), 2.07 (s, 3 H, CH_3), 2.54 (t, 2 H, CH_2), 2.64 (t, 2 H, J = 7.5 Hz, CH_2), 3.88 (s, 3 H, OCH_3), 5.90 (br, 2 H, 2-NH_2, exch), 7.32 (d, J = 8.0 Hz, 2 H, Ar), 7.86 (d, J = 8.0 Hz, 2 H, Ar),10.1 (s, 1 H, 3-NH, exch), 10.53 (s, 1 H, 7-NH, exch). Anal. Calcd. For: $C_{18}H_{20}N_4O_3 \cdot 0.1\,H_2O$: C, 63.18; H, 5.95; N, 16.37. Found: C, 62.99; H, 5.92; N, 16.35.

Ethyl 5-(2-(2-amino-6-methyl-4-oxo-4,7-dihydro-3*H*-pyrrolo[2,3-*d*]pyrimidin-5-yl)ethyl)thiophene-2-carboxylate (19c)

A mixture of **18c** (500 mg, 1.38 mmol) in diphenyl ether (25 mL) was stirred under N_2 and stirred at 240 ˚C for 8 h. After cooling to room temperature, hexanes (100 mL) was added and the precipitated solid was collected by filtration. The resulting solid was suspended in MeOH (50 mL) and silica gel (5 g) was added and the solvent was evaporated to afford a plug. The silica gel plug obtained was loaded onto a silica gel column and eluted with a gradient of 5-10% MeOH in $CHCl_3$ to obtain **19c** (315 mg, 66%) as a yellow powder. TLC R_f 0.67 ($CHCl_3$/MeOH, 5:1 with 2 drops of 25% NH_4OH); mp 223.7 ˚C; ^{1}H NMR (400 MHz, DMSO-d_6): δ 1.27 (t, J = 7.1 Hz, 3 H, $COOCH_2\underline{CH_3}$), 1.93 (s, 3 H, Ar-CH_3), 2.81 (t, J = 7.3 Hz, 2 H, CH_2), 3.15 (t, J = 7.3 Hz, 2 H, CH_2), 4.24 (q, J = 7.1 Hz, 2 H, $COO\underline{CH_2}CH_3$), 5.97 (s, 2 H, 2-NH_2, exch), 6.87 (d, J = 3.7 Hz, 1 H, Ar), 7.59 (d, J = 3.7 Hz, 1 H, Ar), 10.12 (s, 1 H, 3-NH, exch), 10.58 (s, 1 H, 7-NH, exch).

Methyl 4-(2-(2-amino-6-methyl-4-oxo-4,7-dihydro-3*H*-pyrrolo[2,3-*d*]pyrimidin-5-yl)ethyl)-2-fluorobenzoate (19d)

A mixture of **18d** (1.4 g, 3.9 mmol), in diphenyl ether (20 mL) was stirred and heated at reflux for 6 h. After cooling to room temperature, hexanes were added, and the precipitated powder was collected by filtration and run through a silica column with 5% MeOH in $CHCl_3$ as the eluent to give cyclized **19d** (0.164 g, 0.48 mmol) as a yellow powder in 12% yield. TLC R_f 0.37 ($CHCl_3$/MeOH, 10:1 with 2 drops of 25% NH_4OH); mp 288 ˚C dec; ^{1}H NMR (DMSO-d_6) δ 1.86 (s, 3 H, CH_3), 2.76-2.80 (t, 2 H, CH_2), 2.90-2.94 (t, 2 H, CH_2), 3.83 (s, 3 H, OCH_3), 5.97 (s, 2 H, NH_2, exch.), 7.07-7.11 (m, 2 H, Ar), 7.75-7.79 (t, 1 H, Ar), 10.10 (s, 1 H, NH, exch.), 10.53 (s, 1 H, NH, exch.).

4-(2-(2-Amino-6-methyl-4-oxo-4,7-dihydro-3*H*-pyrrolo[2,3-*d*]pyrimidin-5-yl)ethyl)benzoic acid (20a)

To **19a** (250 mg, 0.77 mmol) was added 1 N NaOH (5 mL) and the resulting mixture was stirred under N_2 at room temperature for 12 h. TLC indicated the disappearance of starting material and the formation of one major polar spot ($CHCl_3$/MeOH). The resulting solution

was cooled in an ice bath, and the pH was adjusted to 3-4 using 1 N HCl. The resulting suspension was chilled in a dry ice/acetone bath and thawed to 4 ˚C overnight in a refrigerator. The precipitate was filtered, washed with cold water, and dried in a desiccator under reduced pressure using P_2O_5 to afford acid **20a** (150 mg, 63%) as a buff colored powder. TLC R_f 0.1 ($CHCl_3$/MeOH, 5:1); mp 194 ˚C.[1] ^{1}H NMR (400 MHz, DMSO-d_6): δ 1.84 (s, 3 H, CH_3), 2.75-2.79 (t, *J*=7.29, 7.29 Hz, 2 H, Ar-CH_2), 2.87-2.91 (m, 2 H, Ar-CH_2), 6.3 (s, 2 H, 2-NH_2, exch), 7.23-7.25 (d, *J* = 8.16 Hz, 2 H, Ar), 7.81-7.83 (d, *J* = 8.13 Hz, 2 H, Ar), 10.4 (s, 1 H, 3-NH, exch), 10.7 (s, 1 H, 7-NH, exch). ^{1}H NMR matches with the reported NMR.[1]

4-(3-(2-Amino-6-methyl-4-oxo-4,7-dihydro-3*H*-pyrrolo[2,3-*d*]pyrimidin-5-yl)propyl)benzoic acid (20b)

To a solution of **19b** (0.45 g, 1.44 mmol) in 2:1 MeOH:DMSO (30 mL) was added 1 N NaOH (5 mL) and the mixture stirred under N_2 at room temperature for 24 h to complete the reaction. The reaction mixture was evaporated to dryness under reduced pressure. The residue was dissolved in distilled water (10 mL) and the solution was filtered through a celite pad and washed with water (5 mL). The filtrate was cooled in an ice bath and the pH adjusted to 4.0 by dropwise addition of 1 N HCl. The resulting suspension was filtered, washed with a small amount of cold water and ethyl ether and dried in vacuo to afford **20b** (0.35 g, 82%) as a buff colored solid; mp 220 ˚C dec; ^{1}H NMR (300 MHz, DMSO-d_6): δ 1.89 (t, 2 H, CH_2), 2.61 (m, 4 H, $\underline{CH_2}CH_2\underline{CH_2}$), 5.91 (s, 2 H, 2-$NH_2$, exch), 7.29 (d, *J* = 7.2 Hz, 2 H, Ar), 7.84 (d, *J* = 7.2 Hz, 2 H, Ar), 10.02 (s, 1 H, 3-NH, exch), 10.53 (s, 1 H, 7-NH, exch) 12.73 (br, 1 H, COOH, exch). HRMS (EI): Calculated for $C_{17}H_{18}N_4O_3$, *m/z* = 326.1379, found *m/z* = 326.1380.

5-(2-(2-Amino-6-methyl-4-oxo-4,7-dihydro-3*H*-pyrrolo[2,3-*d*]pyrimidin-5-yl)ethyl)thiophene-2-carboxylic acid (20c)

To **19c** (270 mg, 0.78 mmol) was added 1 N NaOH (5 mL) and the resulting mixture was stirred under N_2 at 40 ˚C for 8 h. TLC indicated the disappearance of starting material and the formation of one major polar spot ($CHCl_3$/MeOH). The resulting solution was cooled in an ice bath, and the pH was adjusted to 3-4 using 1 N HCl. The resulting suspension was allowed to chill at 4 ˚C overnight in a refrigerator. The precipitate was then filtered, washed with cold water, and dried in a desiccator under reduced pressure using P_2O_5 to afford acid **20c** (235 mg, 94%) as a buff colored powder. mp 180.4 ˚C. ^{1}H NMR (500 MHz, DMSO-d_6): δ 1.92 (s, 3 H, Ar-CH_3), 2.80 (t, *J* = 7.3 Hz, 2 H, CH_2), 3.13 (t, *J* = 7.3 Hz, 2 H, CH_2), 5.99 (br, 2 H, 2-NH_2, exch), 6.83 (dd, *J* = 3.8, 0.9 Hz, 1 H, Ar), 7.52 (d, *J* = 3.7 Hz, 1 H, Ar), 10.14 (s, 1 H, 3-NH, exch), 10.58 (s, 1 H, 7-NH, exch).

4-(2-(2-Amino-6-methyl-4-oxo-4,7-dihydro-3*H*-pyrrolo[2,3-*d*]pyrimidin-5-yl)ethyl)-2-fluorobenzoic acid (20d)

To **19d** (0.160 g, 0.46 mmol) was added 1 N NaOH (5 mL) and the resulting mixture was stirred under N_2 at room temperature for 12 h. TLC indicated the disappearance of starting material and the formation of one major polar spot ($CHCl_3$/MeOH). The resulting solution was cooled in an ice bath, and the pH was adjusted to 3-4 using 1 N HCl. The resulting suspension was chilled in a dry ice/acetone bath and thawed to 4 ˚C overnight in a refrigerator. The precipitate was filtered, washed with cold water, and dried in a desiccator

under reduced pressure using P_2O_5 to afford acid **20d** (0.132 g, 0.4 mmol) as a buff colored powder in 86% yield. TLC R_f 0.62 ($CHCl_3$/MeOH, 10:1 with 2 drops of glacial acetic acid); mp 155 ˚C dec; ^{1}H NMR (DMSO-d_6) δ 1.87 (s, 3 H, CH_3), 2.75-2.79 (t, 2 H, CH_2), 2.89-2.92 (t, 2 H, CH_2), 5.97 (s, 2 H, NH_2, exch.), 7.03-7.04 (d, 1 H, Ar), 7.06 (s, 1 H, Ar), 7.72-7.76 (t, 1 H, Ar), 10.11 (s, 1 H, NH, exch.), 10.53 (s, 1 H, NH, exch.), 13.05 (s, 1 H, COOH, exch.).

Diethyl (4-(2-(2-amino-6-methyl-4-oxo-4,7-dihydro-3*H*-pyrrolo[2,3-*d*]pyrimidin-5-yl)ethyl)benzoyl)-L-glutamate (21a).

To a solution of **20a** (150 mg, 0.48 mmol) in anhydrous DMF was added 6-chloro-2,4-dimethoxy-1,3,5-triazine (101 mg, 0.58 mmol) and *N*-methylmorpholine (0.06 mL, 0.58 mmol). After the mixture was stirred at room temperature for 2 h, *N*-methylmorpholine (0.06 mL, 0.58 mmol, 1.2 eq) and diethyl L-glutamate hydrochloride (146 mg, 0.72 mmol, 1.5 eq) were added all at once. The mixture was stirred at room temperature for 12 h. TLC showed the formation of one major non-polar spot and disappearance of starting material ($CHCl_3$/MeOH). The reaction mixture was evaporated to dryness under reduced pressure and the residue was dissolved in MeOH. To the solution, celite was added and the solvent was evaporated to afford a plug. The plug was loaded on a silica gel column and flash-chromatographed with $CHCl_3$ followed by gradual increase in polarity to 5% MeOH in $CHCl_3$. The fractions with desired R_f were pooled, evaporated and the residue was dried in vacuo using P_2O_5 to afford the glutamate ester **21a** (100 mg, 42%) as a light pink sticky solid. TLC R_f 0.5 ($CHCl_3$/MeOH, 5:1); ^{1}H NMR (400 MHz, DMSO-d_6): δ 1.15-1.2 (m, 6 H, $COOCH_2\underline{CH_3}$), 1.85 (s, 3 H, Ar-CH_3), 1.96-2.15 (m, 2 H, β-CH_2), 2.42-2.46 (t, *J* = 7.86, 7.86 Hz, 2 H, γ-CH_2) 2.75-2.79 (m, 2 H, Ar-CH_2), 2.87-2.91 (m, 2 H, Ar-CH_2), 4.02-4.16 (m, 4 H, $COO\underline{CH_2}CH_3$), 4.39-4.45 (m, 1 H, α-CH), 5.96 (s, 2 H, 2-NH_2, exch), 7.22-7.24 (d, *J* = 8.28 Hz, 2 H, Ar), 7.75-7.77 (d, *J* = 8.07 Hz, 2 H, Ar), 8.64-8.66 (d, *J* = 7.39 Hz, 1 H, CONH exch), 10.1 (s, 1 H, 3-NH, exch), 10.5 (s, 1 H, 7-NH, exch). ^{1}H NMR matches with the reported NMR.[1]

Diethyl (4-(3-(2-amino-6-methyl-4-oxo-4,7-dihydro-3*H*-pyrrolo[2,3-*d*]pyrimidin-5-yl)propyl)benzoyl)-L-glutamate (21b)

To a solution of **20b** (0.22 g, 0.74 mmol) in anhydrous DMF (18 mL) was added TEA (0.26 mL) and the mixture was stirred under N_2 at room temperature for 5 min. The resulting solution was cooled to 0 ˚C, isobutyl chloroformate (0.26 mL, 1.92 mmol) was added and the mixture was stirred at 0 ˚C for 30 min. At this time TLC ($CHCl_3$/MeOH, 5:1) indicated the formation of the activated intermediate at R_f 0.50 and the disappearance of the starting acid (R_f0.30). Diethyl L-glutamate hydrochloride (0.475 g, 1.92 mmol) was added to the reaction mixture followed immediately by TEA (0.26 mL, 1.92 mmol). The reaction mixture was slowly allowed to warm to room temperature and stirred under N_2 for 12 h. The reaction mixture was then subjected to another cycle of activation and coupling using half the quantities listed above. The reaction mixture was slowly allowed to warm to room temperature and stirred for an additional 24 h. The reaction mixture was then subjected to a third round of activation and coupling using the same quantities as the second round and was stirred for an additional 24 h. TLC showed the formation of one major spot at R_f 0.52 ($CHCl_3$/MeOH, 5:1). The reaction mixture was evaporated to dryness under reduced pressure. The residue was dissolved in MeOH (5 mL) and silica gel (10 g) was added and

the solvent was evaporated to afford a plug. The silica gel plug obtained was loaded onto a silica gel column and eluted with 19:1 $CHCl_3$/ MeOH to obtain **21b** (0.2 g, 61%) as a pale-yellow solid. TLC R_f 0.52 ($CHCl_3$/MeOH, 5:1); mp 190 ˚C dec. ^{1}H NMR (300 MHz, DMSO-d_6): δ 1.13-1.21 (m, 6 H, $COOCH_2\underline{CH_3}$), 1.84 (m, 2 H, β-CH_2), 2.02 (s, 3 H, CH_3), 2.42-2.46 (m, 8 H, $\underline{CH_2CH_2}$ & $\underline{CH_2}CH_2\underline{CH_2}$), 3.05 (m, 4 H, α- & γ-$COO\underline{CH_2}CH_3$), 4.40 (m, 1 H, α-CH), 5.95 (bs, 2 H, 2-NH_2, exch), 7.28 (d, J = 7.0 Hz, 2 H, Ar), 7.79 (d, J = 7.0 Hz, 2 H, Ar), 8.65 (d, J = 7.3 Hz, 1 H, CONH exch), 10.05 (s, 1 H, 3-NH, exch), 10.54 (s, 1 H, 7-NH, exch). Anal. Calcd. For: $C_{26}H_{33}N_5O_6$: C, 61.04; H, 6.50; N, 13.69. Found: C, 61.41; H, 6.60; N, 13.69.

Dimethyl (5-(2-(2-amino-6-methyl-4-oxo-4,7-dihydro-3*H*-pyrrolo[2,3-*d*]pyrimidin-5-yl)ethyl)thiophene-2-carbonyl)-L-glutamate (21c)

To a solution of **20c** (150 mg, 0.47 mmol) in anhydrous DMF was added 6-chloro-2,4-dimethoxy-1,3,5-triazine (109 mg, 0.62 mmol) and *N*-methylmorpholine (0.07 mL, 0.62 mmol). After the mixture was stirred at room temperature for 2 h, *N*-methylmorpholine (0.06 mL, 0.62 mmol) and dimethyl L-glutamate hydrochloride (150 mg, 0.70 mmol) were added all at once. The mixture was stirred at room temperature for 12 h. TLC showed the formation of one major non-polar spot. The reaction mixture was evaporated to dryness under reduced pressure and the residue was dissolved in MeOH. To the solution, silica was added and the solvent was evaporated to afford a plug. The plug was loaded on a silica gel column and flash-chromatographed with $CHCl_3$ followed by gradual increase in polarity to 10% MeOH in $CHCl_3$. The fractions with desired R_f were pooled, evaporated and the residue was dried in vacuo using P_2O_5 to afford the glutamate ester **21c** (67 mg, 30%) as a red brown semi solid. TLC R_f 0.2 ($CHCl_3$/MeOH, 10:1). ^{1}H NMR of **21c** presented additional aliphatic peaks, suggesting presence of a non-polar impurities. In an attempt to purify the compound, it was triturated with n-hexanes (3 x 10 mL) and filtered. However, this led to loss of compound without significant effect on purity. Therefore, it was taken further for the next step.

Diethyl (4-(2-(2-amino-6-methyl-4-oxo-4,7-dihydro-3*H*-pyrrolo[2,3-*d*]pyrimidin-5-yl)ethyl)-2-fluorobenzoyl)-L-glutamate (21d)

To a solution of **20d** (0.115 g, 0.35 mmol) in anhydrous DMF was added 6-chloro-2,4-dimethoxy-1,3,5-triazine (0.73 g, 0.42 mmol) and *N*-methylmorpholine (0.04 mL, 0.42 mmol, 1.2 eq). After the mixture was stirred at room temperature for 2 h, *N*-methylmorpholine (0.04 mL, 0.42 mmol, 1.2 eq) and diethyl L-glutamate hydrochloride (0.125 g, 0.52 mmol, 1.5 eq) were added all at once. The mixture was stirred at room temperature for 12 h. TLC showed the formation of one major non-polar spot and disappearance of starting material ($CHCl_3$/MeOH). The reaction mixture was evaporated to dryness under reduced pressure and the residue was dissolved in MeOH. To the solution, celite was added and the solvent was evaporated to afford a plug. The plug was loaded on a silica gel column and flash-chromatographed with $CHCl_3$ followed by gradual increase in polarity to 5% MeOH in $CHCl_3$. The fractions with desired R_f were pooled, evaporated and the residue was dried in vacuo using P_2O_5 to afford the glutamate ester **21d** (0.140 g, 0.27 mmol) as a light pink sticky solid in 78% yield. TLC R_f 0.48 ($CHCl^-{}_3$/MeOH, 10:1 with 2 drops of glacial acetic acid); mp 155 ˚C dec; ^{1}H NMR (400 MHz, DMSO-d_6): δ 1.16-1.22 (m, 6 H, $COOCH_2\underline{CH_3}$), 1.90 (s, 3 H, Ar-CH_3), 1.94-2.12 (m, 2 H,

β-CH_2), 2.42-2.45 (t, 2 H, γ-CH_2) 2.75-2.79 (m, 2 H, Ar-CH_2), 2.88-2.92 (m, 2 H, Ar-CH_2), 4.03-4.15 (m, 4 H, COO$\underline{CH_2}$$CH_3$), 4.39-4.45 (m, 1 H, α-CH), 5.97 (s, 2 H, 2-NH_2, exch.), 7.05 (s, 1 H, Ar), 7.07 (d, 1 H, Ar), 7.46-7.50 (t, 1 H, Ar), 8.56-8.58 (d, 1 H, CONH exch.), 10.11 (s, 1 H, 3-NH, exch.), 10.53 (s, 1 H, 7-NH, exch.)

Methyl 2-fluoro-4-(4-hydroxybut-1-yn-1-yl)benzoate (23)
To a 20-mL vial for microwave reaction, was added a mixture of $PdCl_2$ (182.15 mg, 1.03 mmol), PPh_3 (269.44 mg, 1.03 mmol), TEA (6.5 g, 64.20 mmol methyl 4-bromo-2-fluorobenzoate **11d** (**Z** = Br) (1.5 g, 6.42 mmol) and anhydrous acetonitrile (8 mL). To the stirred mixture, were added CuI (195.64 mg, 1.03 mmol), and but-3-yn-1-ol **22** (450 mg, 6.42 mmol), and the vial was sealed and put into the microwave reactor at 100 °C for 1 h. TLC showed a new spot with Rf = 0.62 (hexane/ethyl acetate, 1:1). This reaction was repeated two more time with similar outcome. The three reaction mixtures were combined and the solvent was evaporated. Then methanol and celite (10 g) were added and the solvent was evaporated under reduced pressure. The resulting plug was subjected to flash chromatography in ethyl acetate/hexane system. The desired fraction (TLC) was collected and the solvent was evaporated under reduced pressure to afford **23** (2.7 g, yield 63%) as a brown semisolid; TLC R_f 0.62 (hexane/ethyl acetate, 1:1); ^{1}H NMR (400 MHz, $CDCl_3$): δ 1.85-1.88 (t, J = 6.0 Hz, 1 H), 2.72-2.75 (t, J = 6.3 Hz, 2 H), 3.84-3.88 (q, J = 5.8 Hz, 2 H), 7.17-7.26 (m, 2 H), 3.95 (s, 3 H), 7.87-7.91 (t, J = 7.8 Hz, 1 H).

Methyl 2-fluoro-4-(4-hydroxybutyl)benzoate (24)
To a Parr flask was added **23** (2 g, 9 mmol), 10% Pd/C (2 g), and MeOH (100 mL). Hydrogenation was carried out at 55 psi for 9 h at rt. The reaction mixture was filtered through celite, washed with MeOH (100 mL) and concentrated under reduced pressure to give 1.93 g (95%) of **24** as a colorless liquid; TLC R_f 0.56 (hexane/ethyl acetate, 1:1); ^{1}H NMR (400 MHz, $CDCl_3$): δ 1.43 (s, 1 H), 1.58-1.78 (m, 4 H), 2.68-2.72 (t, J = 7.6 Hz, 2 H), 3.67-3.70 (t, J = 6.3 Hz, 2 H), 3.93 (s, 3 H), 6.96-7.05 (m, 2 H), 7.85-7.88 (t, J = 7.8 Hz, 1 H).

Methyl 2-fluoro-4-(4-oxobutyl)benzoate (25)
To a stirred solution of Dess-Martin periodinane (4.34 g, 10.24 mmol) in CH_2Cl_2 (8 mL), methyl 2-fluoro-4-(4-hydroxybutyl)benzoate, **24** (1.93 g, 8.53 mmol) in anhydrous CH_2Cl_2 (8 mL) was added at 0 °C. Stirring was continued for 3 h and the mixture was allowed to warm to room temperature. When the reaction was complete, excess oxidant was destroyed by the addition of 1N NaOH. After 30 min of vigorous stirring, the mixture was diluted with ethyl acetate (10 mL), the organic layer was separated, and the aqueous layer was extracted with ethyl acetate (2 x 10 mL). The combined extracts were washed with brine, dried with $MgSO_4$, and concentrated to afford **25** (853 mg, yield 45%) as a colorless liquid; TLC R_f 0.68 (hexane/ethyl acetate 1:1); ^{1}H NMR (400 MHz, $CDCl_3$): δ 1.95-2.03 (p, J = 7.3 Hz, 2 H), 2.49-2.53 (td, J = 1.4, 7.3 Hz, 2 H), 3.94 (s, 3 H), 2.69-2.73 (m, 2 H), 6.97-7.05 (m, 2 H), 7.87- 7.91 (t, J = 7.8 Hz, 1 H), 9.80-9.81 (t, J = 1.4 Hz, 1 H).

Methyl 4-(3-bromo-4-oxobutyl)-2-fluorobenzoate (26)

To 1,4-dioxane (618.17 mg, 600.16 mmol) dissolved in CH_2Cl_2 (3 ml), Br_2 (640.71 mg, 205.36 mmol) was added dropwise and the mixture was stirred for 10 min, and diluted with CH_2Cl_2 (3 ml). This solution was added dropwise to an ice-cool solution of **25** (899 mg, 4.01 mmol) in CH_2Cl_2 (5 ml) over 3 h under N_2. The mixture was stirred for 0.5 h, then a solution of Na_2CO_3 in water (10 ml) was added. After being stirred for 1h, the reaction mixture was extracted with $CHCl_3$ (2 x 10 ml). TLC was run and a new non-polar spot was identified. The compound **26** was unstable, so, it was immediately used for the next step.

Methyl 4-(2-(2-amino-4-oxo-4,7-dihydro-3*H*-pyrrolo[2,3-*d*]pyrimidin-5-yl)ethyl)-2-fluorobenzoate (27)

To a solution of 2,6-diamino-4-oxopyrimidine (558.35 mg, 4.43 mmol) and sodium acetate (660.32 mg, 8.05 mmol) in water (10 mL) and MeOH (10 mL) was added α-bromo aldehyde **26** (4.02 mmol). The reaction mixture was stirred at 45 °C for 12 h. TLC showed the disappearance of starting materials and the formation of one major spot at R_f 0.54 ($CHCl_3$/MeOH, 5:1). After evaporation of the solvent, MeOH (10 mL) was added followed by celite (4 g). Evaporation of the solvent afforded a plug, which was eluted by combiflash ($CHCl_3$/MeOH system). Fractions showing R_f 0.54 were pooled and evaporated to afford **27** (608 mg, yield 46% over two steps) as a pink powder; TLC R_f 0.54 ($CHCl_3$/MeOH, 5:1); mp 251 °C dec; ^1H NMR (DMSO- d_6): δ 2.84-2.88 (dd, J = 6.2, 9.2 Hz, 2 H), 2.99-3.02 (dd, J = 6.3, 9.1 Hz, 2 H), 3.83 (s, 3 H), 6.03 (s, 2 H), 6.31- 6.33 (d, J = 2.1 Hz, 1 H), 7.14-7.18 (m, 2 H), 7.77-7.81 (t, J = 8.0 Hz, 1 H), 10.19 (s, 1 H), 10.65 (s, 1 H). Anal. calculated for ($C_{16}H_{15}FN_4O_3 \cdot 0.46\ H_2O$): C, 56.74; H, 4.74; N, 16.54; F, 5.60; Found: C, 56.75; H, 4.55; N, 16.48; F, 5.48.

4-(2-(2-Amino-4-oxo-4,7-dihydro-3*H*-pyrrolo[2,3-*d*]pyrimidin-5-yl)ethyl)-2-fluorobenzoic acid (28)

To **27** (458 mg, 1.39 mmol) was added 1 N NaOH (3 mL). The resulting mixture was stirred at room temperature for 3 h. TLC indicated the disappearance of starting material and the formation of one major spot at R_f 0.45 ($CHCl_3$/MeOH, 5:1). To this was added distilled water (3 mL). The solution was cooled in an ice bath, and the pH was adjusted 3 to 4 using 1 N HCl. The resulting suspension was thawed to 4 °C overnight in a refrigerator. The precipitate was filtered, washed with cold water, and dried in a desiccator under reduced pressure using P_2O_5 to afford **28** (420 mg, yield 96%) as a light pink powder; mp 274 °C dec; TLC R_f 0.45 ($CHCl_3$/MeOH, 5:1); ^1H NMR (DMSO- d_6): δ 2.84-2.88 (t, J = 7.7 Hz, 2 H), 2.97-3.01 (t, J = 7.6 Hz, 2 H), 6.04 (s, 2 H), 6.31-6.33 (d, J = 2.1 Hz, 1 H), 7.11-7.13 (d, J = 9.8 Hz, 2 H), 7.74-7.78 (t, J = 7.9 Hz, 1 H), 10.19 (s, 1 H), 10.65 (s, 1 H). MS (ESI): m/z calcd for $C_{15}H_{14}FN_4O_3$ $[M + H]^+$ 317.1044; found, 317.1087.

Diethyl (4-(2-(2-amino-4-oxo-4,7-dihydro-3*H*-pyrrolo[2,3-*d*]pyrimidin-5-yl)ethyl)-2-fluorobenzoyl)-L-glutamate (29)

In 20 mL DMF, **28** (300 mg, 0.948 mmol) was added. Then 2-chloro-4,6-dimethoxy-1,3,5-triazine (199.83 mg, 1.14 mmol), *N*-methylmorpholine (239.85 mg, 2.37 mmol) and L-glutamate diethyl ester hydrochloride (293.01 mg, 1.44 mmol) were added in the stirred solution. This was stirred at room temperature for 12 hrs. TLC showed formation of a major new spot at R_f 0.60 ($CHCl_3$/MeOH, 5:1). The reaction mixture was evaporated to dryness under reduced pressure. The residue was dissolved in MeOH, celite added and

solvent evaporated to make a plug. From column run in combiflash ($CHCl_3$/MeOH), fractions that showed the desired single spot at R_f 0.60 were pooled and evaporated to dryness to afford **29** (280 mg, yield 59%) as a grey powder; mp 228 °C dec; TLC R_f 0.60 ($CHCl_3$/MeOH, 5:1); ^{1}H NMR (400 MHz, DMSO- d_6): δ 1.14-1.23 (dt, J = 7.1, 11.0 Hz, 6 H), 1.90-2.13 (m, J = 7.5, 13.1 Hz, 2 H), 2.41-2.46 (td, J = 2.5, 6.9, 7.4 Hz, 2 H), 2.83-2.88 (dd, J = 6.3, 9.1 Hz, 2 H), 2.96-3.02 (dd, J = 6.1, 9.1 Hz, 2 H), 4.03-4.08 (q, J = 7.1 Hz, 2 H), 4.08-4.15 (qd, J = 2.1, 7.2 Hz, 2 H), 4.38- 4.45 (ddd, J = 5.0, 7.4, 9.5 Hz, 1 H), 6.06 (s, 2 H), 6.32-6.34 (d, J = 2.0 Hz, 1 H), 7.10-7.13 (m, 2 H), 7.47-7.51 (t, J = 7.7 Hz, 1 H), 8.57-8.59 (dd, J = 2.0, 7.5 Hz, 1 H), 10.17 (s, 1 H), 10.64 (d, J = 2.2 Hz, 1 H). MS (ESI): m/z calcd for $C_{24}H_{29}FN_5O_6$ [M + H]$^+$ 502.2096; found, 502.2172.

(4-(2-(2-Amino-4-oxo-4,7-dihydro-3*H*-pyrrolo[2,3-*d*]pyrimidin-5-yl)ethyl)-2-fluorobenzoyl)-L-glutamic acid (4)

To **29** (122 mg, 0.369 mmol) was added 1 N NaOH (2 mL). The resulting mixture was stirred at room temperature for 3 h. TLC indicated the disappearance of starting material and the formation of one very polar major spot. To this was added distilled water (2 mL). The solution was cooled in an ice bath, and the pH was adjusted 3 to 4 using 1 N HCl. The resulting suspension was thawed to 4 °C overnight in a refrigerator. The precipitate was filtered, washed with cold water, and dried in a desiccator under reduced pressure using P_2O_5 to afford **4** (65 mg, yield 56%) as a light green powder; TLC R_f 0.22 ($CHCl_3$/MeOH, 5:1 in acidic condition); mp 231 °C; ^{1}H NMR (DMSO-d_6): δ 1.91-2.09 (m, 2 H), 2.33-2.35 (d, J = 8.2 Hz, 2 H), 2.84-2.88 (m, 2 H), 2.97-3.01 (dd, J = 6.0, 9.2 Hz, 2 H), 4.39 (t, J = 11.1 Hz, 1 H), 6.04 (s, 2 H), 6.32-6.34 (d, J = 2.0 Hz, 1 H), 7.10-7.12 (d, J = 9.6 Hz, 2 H), 7.50-7.54 (t, J = 7.7 Hz, 1 H), 8.42-8.44 (m, 1 H), 10.18 (s, 1 H), 10.64-10.65 (d, J = 2.3 Hz, 1 H). ^{13}C NMR (125 MHz, DMSO-d_6) δ 174.36, 173.59, 164.41, 159.79, 159.72 (d, $^1J_{CF}$ = 249.8 Hz), 152.70, 151.89, 148.72 (d, $^3J_{CF}$ = 8.0 Hz), 130.40 (d, $^3J_{CF}$ = 3.1 Hz), 124.88 (d, $^4J_{CF}$ = 3.2 Hz), 121.13 (d, $^2J_{CF}$ = 13.6 Hz), 117.79, 116.19 (d, $^2J_{CF}$ = 21.9 Hz), 114.03, 99.15, 52.30, 36.19, 30.61, 28.16, 26.38. MS (ESI): m/z calcd for $C_{20}H_{21}FN_5O_6$ [M + H]$^+$ 446.1470; found, 446.1465. HPLC purity: 95.74% (260 nm).

(4-(2-(2-Amino-6-methyl-4-oxo-4,7-dihydro-3*H*-pyrrolo[2,3-*d*]pyrimidin-5-yl)ethyl)benzoyl)-L-glutamic acid (5).

To **21a** (60 mg, 0.12 mmol), was added 1 N NaOH (3 mL) and the resulting mixture was stirred at room temperature for 1 h. TLC indicated the disappearance of starting material and the formation of one major spot at the origin ($CHCl_3$/MeOH). The solution was cooled in an ice bath, and the pH was adjusted to 3-4 using 1 N HCl. The resulting suspension was chilled in a dry ice/acetone bath and thawed to 4 ˚C overnight in a refrigerator. The precipitate was filtered, washed with cold water, and dried in a desiccator under reduced pressure using P_2O_5, to afford **5** (40 mg, 75%) as a buff colored powder. mp 164.6 ˚C. ^{1}H NMR (400 MHz, DMSO-d_6): δ 1.85 (s, 3 H, Ar-CH_3), 1.94-2.11 (m, 2 H, β-CH_2), 2.33-2.38 (t, J = 7.86, 7.86 Hz, 2 H, γ-CH_2) 2.75-2.78 (m, 2 H, Ar-CH_2), 2.86-2.9 (m, 2 H, Ar-CH_2), 4.35-4.42 (m, 1 H, α-CH), 5.97 (s, 2 H, 2-NH_2, exch), 7.2-7.23 (d, J = 8.25 Hz, 2 H, Ar), 7.75-7.77 (d, J = 8.12 Hz, 2 H, Ar), 8.53-8.55 (d, J = 7.64 Hz, 1 H, CONH exch), 10.11 (s, 1 H, 3-NH, exch), 10.5 (s, 1 H, 7-NH, exch). MS (ESI): m/z calcd for $C_{21}H_{24}N_5O_6$ [M + H]$^+$ 442.1721; found, 442.1721. Anal. Calcd for $C_{21}H_{23}N_5O_6 \cdot 0.95\ H_2O$: C, 54.99; H, 5.47; N, 15.27. Found: C, 55.04; H, 5.42; N, 15.09. ^{1}H NMR matches with the reported NMR.[1]

(4-(3-(2-Amino-6-methyl-4-oxo-4,7-dihydro-3*H*-pyrrolo[2,3-*d*]pyrimidin-5-yl)propyl)benzoyl)-L-glutamic acid (6)

To a solution of **21b** (0.12 g, 0.25 mmol) in THF-H_2O (2:1, 20 mL) was added 1 N NaOH (5 mL). After 24 h of stirring at room temperature, the reaction mixture was concentrated under reduced pressure, and the residual aqueous solution was acidified to pH 4 with 1 N HCl. The precipitate was collected by filtration, washed with water, and dried in vacuo to give 6 (0.08 g, 80%) as an off-white solid. mp 210 ˚C dec. ^{1}H NMR (300 MHz, DMSO-d_6): δ 1.86 (m, 2 H, CH_2), 2.09 (s, 3 H, CH_3), 2.32(t, J = 7.1 Hz, 2 H, 4- $\underline{CH_2}CH_2$), 2.52-2.88 (m, 6 H, $\underline{CH_2}CH_2$ & $\underline{CH_2}CH_2\underline{CH_2}$), 4.38 (m, 1 H, α-CH), 5.91 (bs, 2 H, 2-NH_2, exch), 7.27 (d, J = 7.2 Hz, 2 H, Ar), 7.84 (d, J = 7.2 Hz, 2 H, Ar), 8.53 (d, J = 7.4 Hz, 1 H, CONH exch), 10.03 (s, 1 H, 3-NH, exch), 10.53 (s, 1 H, 7-NH, exch). MS (ESI): m/z calcd for $C_{22}H_{26}N_5O_6$ $[M + H]^+$ 456.1878; found, 456.1876. Anal. Calcd for $C_{22}H_{25}N_5O_6$: C, 58.01; H, 5.53; N, 15.38. Found: C, 58.18; H, 5.35; N, 15.47.

(5-(2-(2-Amino-6-methyl-4-oxo-4,7-dihydro-3*H*-pyrrolo[2,3-*d*]pyrimidin-5-yl)ethyl)thiophene-2-carbonyl)-L-glutamic acid (7)

To **21c** (58 mg, 0.12 mmol), was added 1 N NaOH (3 mL) and the resulting mixture was stirred at room temperature for 12 h. TLC indicated the disappearance of starting material and the formation of one major spot at the origin ($CHCl_3$/MeOH). The solution was cooled in an ice bath, and the pH was adjusted to 3-4 using 1 N HCl. The resulting suspension was chilled in a dry ice/acetone bath and thawed to 4 ˚C overnight in a refrigerator. The precipitate was filtered, washed with cold water, and dried. Analytical HPLC indicated presence of a polar impurity. The compound was then triturated with H_2O (3 x 50 mL), filtered, and dried in a desiccator under reduced pressure using P_2O_5, to afford **5** (10 mg, 19%) as a buff colored powder. mp 192.2 ˚C. ^{1}H NMR (400 MHz, DMSO-d_6): δ 1.94 (s, 3 H, Ar-CH_3), 2.08 – 1.90 (m, 2 H, β-CH_2), 2.33 (t, J = 7.5 Hz, 3 H, γ-CH_2), 2.79 (t, J = 7.4 Hz, 2 H, CH_2), 3.10 (t, J = 7.4 Hz, 2 H, CH_2), 4.33 (dd, J = 15.2, 7.3 Hz, 2 H, α-CH), 5.97 (s, 2 H, 2-NH_2, exch), 6.80 (d, J = 3.7 Hz, 1 H, Ar), 7.65 (d, J = 3.8 Hz, 1 H, Ar), 8.48 (d, J = 7.8 Hz, 1 H, CONH exch), 10.11 (s, 1 H, 3-NH, exch), 10.57 (s, 1 H, 7-NH, exch). MS (ESI): m/z calcd for $C_{19}H_{22}N_5O_6S$ $[M + H]^+$ 448.1290; found, 448.1283. HPLC purity: 95.58% (290 nm).

(4-(2-(2-Amino-6-methyl-4-oxo-4,7-dihydro-3*H*-pyrrolo[2,3-*d*]pyrimidin-5-yl)ethyl)-2-fluorobenzoyl)-L-glutamic acid (8)

To **21d** (0.135 g, 0.26 mmol), was added 1 N NaOH (5 mL) and the resulting mixture was stirred at room temperature for 1 h. TLC indicated the disappearance of starting material and the formation of one major spot at the origin ($CHCl_3$/MeOH). The solution was cooled in an ice bath, and the pH was adjusted to 3-4 using 1 N HCl. The resulting suspension was chilled in a dry ice/acetone bath and thawed to 4 ˚C overnight in a refrigerator. The precipitate was filtered, washed with cold water, and dried in a desiccator under reduced pressure using P_2O_5, to afford **8** (0.095 mg, 0.20 mmol) as a buff colored powder in 79% yield. mp 227 ˚C; ^{1}H NMR (400 MHz, DMSO-d_6): δ 1.90 (s, 3 H, Ar-CH_3), 1.88-2.12 (m, 2 H, β-CH_2), 2.33-2.37 (t, 2 H, γ-CH_2) 2.75-2.79 (t, 2 H, Ar-CH_2), 2.88-2.91 (t, 2 H, Ar-CH_2), 4.36-4.41 (m, 1 H, α-CH), 5.97 (s, 2 H, 2-NH_2, exch.), 7.04-7.07 (d, 2 H, ArH), 7.49-7.52 (t, 1 H, ArH), 8.40-8.43 (dd, 1 H, CONH exch.), 10.10 (s, 1 H, 3-NH, exch.), 10.53 (s, 1

H, 7-NH, exch.), 12.42 (s, br, 2 H, COOH, exch.). MS (ESI): m/z calcd for $C_{21}H_{23}FN_5O_6$ $[M + H]^+$ 460.1627; found, 460.1620. Anal. Calcd for $C_{21}H_{22}FN_5O_6 \cdot 1.0\ H_2O$: C, 52.83; H, 5.07; F, 3.98; N, 14.67. Found: C, 52.67; H, 5.09; F, 3.97; N, 14.62.

Molecular modeling protocol. Compounds **1** to **8** were prepared using LigPrep[3] module in Maestro (Schrödinger). The proteins of interest (PDBs: 5IZQ, 4KN2, 7BC7, 8GOF) were prepared using protein preparation workflow[4] in Maestro. Subsequently the compounds were docked in the proteins using the induced fit docking[5] module in Maestro. Default settings were utilized for all three actions. The docked structures of the des- and 6-methyl compounds in a particular protein were examined for relevant, top-scoring docked poses. The selected docked structures were then superimposed using the C-alpha atoms of the protein.

Conformational search for energy minimized conformations: All the compounds designed were constructed in Maestro, and prepared using Ligprep. The Schrödinger molecular modeling package was used to generate OPLS3e force field parameters for each compound. MacroModel[6] was used for search of energy minimized conformations. The mixed torsions and low-mode conformational search (LMCS) was chosen for our calculations. Structures with energies of more than 5 kcal/mole above the lowest-energy conformation were discarded.

Reagents for biological studies. Leucovorin [(6R,S) 5-formyl tetrahydrofolate] and methotrexate (MTX) was provided by the Drug Development Branch, National Cancer Institute, Bethesda, MD. Other chemicals were obtained from commercial sources in the highest available purities. Pemetrexed (PMX; **1**) [N-{4-[2- (2-amino-3,4-dihydro-4-oxo-7H-pyrrolo[2,3-d]pyrimidin-5-yl)ethyl]benzoyl-L-glutamic acid] (Alimta) was purchased from LC Laboratories (Woburn, MA). The syntheses of compounds **2** and **3** were previously described.[7, 8] Additional chemicals were purchased from commercial sources in the highest available purities.

Cell Culture. The reduced folate carrier (RFC), proton-coupled folate transporter (SLC46A1; PCFT) and folate receptor (FR) α- and β-null Chinese hamster ovary (CHO) cell line MTXRIIOua^{R}2-4[9] was a gift from Dr. Wayne Flintoff (University of Western Ontario, London, ON). Four sublines were derived from the R2 subline by transfection with either RFC, PCFT, FRα, or FRβ, giving rise to PC43-10 (expresses human RFC),[10] R2/PCFT4 (expresses human PCFT),[11] RT16 (expresses human FRα), [12] and D4 (expresses FRβ) cells. [12] Isogenic CHO sublines were routinely cultured in α-minimal essential medium (α-MEM) supplemented with 100 units/mL penicillin/100 µg/mL streptomycin, 2 mM *L*-glutamine and 10% bovine calf serum (Sigma-Aldrich). Transfected sublines of R2 cells were cultured in the presence of 1.5 mg/ml G418.

KB nasopharyngeal carcinoma cells were obtained from the American Type Culture Collection (Manassas, VA) and IGROV1 (NCI-IGROV1) (passage 5) ovarian adenocarcinoma cells[13] were obtained from the Division of Cancer Treatment and Diagnosis, National Cancer Institute (Frederick, MD). Normal ovary cells immortalized with SV40 (IOSE 7576)[14] were obtained from the Canadian OvCaRe Cell Bank

(Vancouver, B.C). IGROV1, KB and IOSE 7576 cells were cultured in complete FF-RPMI with 10% fetal bovine serum and 100 units/mL penicillin/100 µg/mL streptomycin, and 2 mM *L*-glutamine. Cell lines were tested for *Mycoplasma* by PCR using a *Mycloplasma* testing kit (Venor™ GeM *Mycoplasma* Detection Kit, Sigma). Frozen stocks were generated from authenticated *Mycoplasma*-free cultures.

For cell proliferation assays, CHO, KB, NCI-IGROV1 and IOSE 7576 cells were plated in 96-well dishes at densities ranging from 2500-5000 cells/well in 200 µL media and treated with a range of inhibitors spanning 0-1000 nM. Experiments with RT16, D4, IGROV1, IOSE 7576 and KB cells used folate-free RPMI 1640 (FF RPMI) media with 10% dialyzed fetal bovine serum and 100 units/mL penicillin/100 µg/mL streptomycin, supplemented with 2 nM leucovorin and 2 mM *L*-glutamine. FR-mediated drug uptake was assessed in parallel incubations including 200 nM folic acid. For R2/PCFT4, PC43-10 and R2 CHO cells, the medium was FF-RPMI supplemented with 10% dialyzed fetal bovine serum, 100 units/mL penicillin/100 µg/mL streptomycin, 25 nM leucovorin and 2 mM *L*-glutamine. Cells were treated over a 96 h period at 37° C with 5% CO_2 and relative cell numbers quantified using the CellTiter-blue cell viability assay (Promega, Madison, WI) and a fluorescence plate reader. Raw data were exported to Excel for analysis and the results were plotted using Graphpad Prism 6.0. Determinations of IC_{50}s were made corresponding to the drug concentrations that resulted in 50% loss of cell growth. These assays have been previously described.[7, 8, 11, 12, 15-20]

Using KB cells, additional proliferation assays were performed with metabolite protection in folate- and glycine-free RPMI1640 with 10% dialyzed fetal bovine serum, 2 mM *L*-glutamine and antibiotics with 2 nM leucovorin to identify the targeted pathway and enzyme(s). [7, 8, 11, 12, 15-20] Inhibitory effects of the novel analogs on *de novo* thymidylate biosynthesis and *de novo* purine nucleotide biosynthesis were tested by incubating drugs with thymidine (10 µM) and adenosine (60 µM), respectively. Incubation with glycine (130 µM) was used to assess the potential involvement of mitochondrial C1 targeted agents.[15] To identify multiple metabolic targets, metabolite additions were added in combination.[7, 8, 11, 12, 15-19] Finally, to distinguish targeting between the folate-dependent purine biosynthetic enzymes glycinamide ribonucleotide formyltransferase (GARFTase) and 5-aminoimidazole-4-carboxamide (AICA) ribonucleotide formyltransferase (AICARFTase), co-incubation experiments were performed using AICA hydrochloride (320 µM). [7, 8, 11, 12, 15-19]

Statistics. The biological data reflect at least three experimental replicates. All statistical comparisons were performed using unpaired t-tests. Biological data are presented as mean values +/- standard errors using data without transformation.

References

1. Taylor, E. C.; Hu, B., A Fischer-indole approach to pyrrolo[2,3-*d*]pyrimidines. *Heterocycles* **1996,** *43*, 323-338.

2. C. Taylor, E.; Wang, Y., Synthesis of 7-Methyl Derivatives of 5,10-Dideaza-5,6,7,8-tetrahydrofolic Acid (DDATHF), 5,10-Dideaza-5,6,7,8-tetrahydrohomofolic Acid (HDDATHF), and LY254155. *Heterocycles* **1998,** *48*.
3. **Schrödinger Release 2023-1**: LigPrep, S., LLC, New York, NY, 2021.
4. **Schrödinger Release 2023-1**: Protein Preparation Wizard; Epik, S., LLC, New York, NY, 2021; Impact, Schrödinger, LLC, New York, NY; Prime, Schrödinger, LLC, New York, NY, 2021.
5. **Schrödinger Release 2023-1**: Induced Fit Docking protocol; Glide, S., LLC, New York, NY, 2021; Prime, Schrödinger, LLC, New York, NY, 2021.
6. **Schrödinger Release 2023-1**: MacroModel, S., LLC, New York, NY, 2021.
7. Mitchell-Ryan, S.; Wang, Y.; Raghavan, S.; Ravindra, M. P.; Hales, E.; Orr, S.; Cherian, C.; Hou, Z.; Matherly, L. H.; Gangjee, A., Discovery of 5-substituted pyrrolo[2,3-d]pyrimidine antifolates as dual acting inhibitors of glycinamide ribonucleotide formyltransferase and 5-aminoimidazole-4-carboxamide ribonucleotide formyltransferase in de novo purine nucleotide biosynthesis: implications of inhibiting 5-aminoimidazole-4-carboxamide ribonucleotide formyltransferase to AMPK activation and anti-tumor activity. *Journal of medicinal chemistry* **2013,** *56*, 10016-32.
8. Wang, Y.; Mitchell-Ryan, S.; Raghavan, S.; George, C.; Orr, S.; Hou, Z.; Matherly, L. H.; Gangjee, A., Novel 5-substituted pyrrolo[2,3-d]pyrimidines as dual inhibitors of glycinamide ribonucleotide formyltransferase and 5-aminoimidazole-4-carboxamide ribonucleotide formyltransferase and as potential antitumor agents. *Journal of medicinal chemistry* **2015,** *58*, 1479-93.
9. Flintoff, W. F.; Nagainis, C. R., Transport of methotrexate in Chinese hamster ovary cells: a mutant defective in methotrexate uptake and cell binding. *Arch Biochem Biophys* **1983,** *223*, 433-40.
10. Wong, S. C.; Proefke, S. A.; Bhushan, A.; Matherly, L. H., Isolation of human cDNAs that restore methotrexate sensitivity and reduced folate carrier activity in methotrexate transport-defective Chinese hamster ovary cells. *J Biol Chem* **1995,** *270*, 17468-75.
11. Deng, Y.; Zhou, X.; Kugel Desmoulin, S.; Wu, J.; Cherian, C.; Hou, Z.; Matherly, L. H.; Gangjee, A., Synthesis and biological activity of a novel series of 6-substituted thieno[2,3-d]pyrimidine antifolate inhibitors of purine biosynthesis with selectivity for high affinity folate receptors over the reduced folate carrier and proton-coupled folate transporter for cellular entry. *Journal of medicinal chemistry* **2009,** *52*, 2940-51.
12. Deng, Y.; Wang, Y.; Cherian, C.; Hou, Z.; Buck, S. A.; Matherly, L. H.; Gangjee, A., Synthesis and discovery of high affinity folate receptor-specific glycinamide ribonucleotide formyltransferase inhibitors with antitumor activity. *Journal of medicinal chemistry* **2008,** *51*, 5052-63.
13. Benard, J.; Da Silva, J.; De Blois, M. C.; Boyer, P.; Duvillard, P.; Chiric, E.; Riou, G., Characterization of a human ovarian adenocarcinoma line, IGROV1, in tissue culture and in nude mice. *Cancer research* **1985,** *45*, 4970-9.
14. Karve, T. M.; Preet, A.; Sneed, R.; Salamanca, C.; Li, X.; Xu, J.; Kumar, D.; Rosen, E. M.; Saha, T., BRCA1 regulates follistatin function in ovarian cancer and human ovarian surface epithelial cells. *PLoS One* **2012,** *7*, e37697.
15. Dekhne, A. S.; Shah, K.; Ducker, G. S.; Katinas, J. M.; Wong-Roushar, J.; Nayeen, M. J.; Doshi, A.; Ning, C.; Bao, X.; Fruhauf, J.; Liu, J.; Wallace-Povirk, A.; O'Connor, C.; Dzinic, S. H.; White, K.; Kushner, J.; Kim, S.; Huttemann, M.; Polin, L.; Rabinowitz, J. D.; Li, J.; Hou, Z.; Dann, C. E., 3rd; Gangjee, A.; Matherly, L. H., Novel Pyrrolo[3,2-d]pyrimidine Compounds Target

Mitochondrial and Cytosolic One-carbon Metabolism with Broad-spectrum Antitumor Efficacy. *Mol Cancer Ther* **2019,** *18*, 1787-1799.
16. Ravindra, M.; Wilson, M. R.; Tong, N.; O'Connor, C.; Karim, M.; Polin, L.; Wallace-Povirk, A.; White, K.; Kushner, J.; Hou, Z.; Matherly, L. H.; Gangjee, A., Fluorine-Substituted Pyrrolo[2,3- d]Pyrimidine Analogues with Tumor Targeting via Cellular Uptake by Folate Receptor alpha and the Proton-Coupled Folate Transporter and Inhibition of de Novo Purine Nucleotide Biosynthesis. *Journal of medicinal chemistry* **2018,** *61*, 4228-4248.
17. Wang, L.; Cherian, C.; Desmoulin, S. K.; Polin, L.; Deng, Y.; Wu, J.; Hou, Z.; White, K.; Kushner, J.; Matherly, L. H.; Gangjee, A., Synthesis and antitumor activity of a novel series of 6-substituted pyrrolo[2,3-d]pyrimidine thienoyl antifolate inhibitors of purine biosynthesis with selectivity for high affinity folate receptors and the proton-coupled folate transporter over the reduced folate carrier for cellular entry. *Journal of medicinal chemistry* **2010,** *53*, 1306-18.
18. Wang, L.; Kugel Desmoulin, S.; Cherian, C.; Polin, L.; White, K.; Kushner, J.; Fulterer, A.; Chang, M. H.; Mitchell-Ryan, S.; Stout, M.; Romero, M. F.; Hou, Z.; Matherly, L. H.; Gangjee, A., Synthesis, biological, and antitumor activity of a highly potent 6-substituted pyrrolo[2,3-d]pyrimidine thienoyl antifolate inhibitor with proton-coupled folate transporter and folate receptor selectivity over the reduced folate carrier that inhibits beta-glycinamide ribonucleotide formyltransferase. *Journal of medicinal chemistry* **2011,** *54*, 7150-64.
19. Wang, L.; Wallace, A.; Raghavan, S.; Deis, S. M.; Wilson, M. R.; Yang, S.; Polin, L.; White, K.; Kushner, J.; Orr, S.; George, C.; O'Connor, C.; Hou, Z.; Mitchell-Ryan, S.; Dann, C. E., 3rd; Matherly, L. H.; Gangjee, A., 6-Substituted Pyrrolo[2,3-d]pyrimidine Thienoyl Regioisomers as Targeted Antifolates for Folate Receptor alpha and the Proton-Coupled Folate Transporter in Human Tumors. *Journal of medicinal chemistry* **2015,** *58*, 6938-59.
20. Wilson, M. R.; Hou, Z.; Yang, S.; Polin, L.; Kushner, J.; White, K.; Huang, J.; Ratnam, M.; Gangjee, A.; Matherly, L. H., Targeting Nonsquamous Nonsmall Cell Lung Cancer via the Proton-Coupled Folate Transporter with 6-Substituted Pyrrolo[2,3-d]Pyrimidine Thienoyl Antifolates. *Mol Pharmacol* **2016,** *89*, 425-34.
